# Milk fat intake, adiposity, and obesity in Canadian children: findings from the prospective Canadian CHILD Cohort Study

**DOI:** 10.1016/j.ajcnut.2025.101186

**Published:** 2026-04-07

**Authors:** Tara Zeitoun, Zheng Hao Chen, David Burgner, Gabbi MacKechnie, Prue Huntington, Toby Mansell, Danielle Longmore, Piushkumar J Mandhane, Elinor Simons, Stuart E Turvey, Padmaja Subbarao, Theo J Moraes, Daniel W Sellen, Kozeta Miliku

**Affiliations:** 1 Department of Nutritional Sciences, University of Toronto, Toronto, Ontario, Canada; 2 Murdoch Children’s Research Institute, The Royal Children’s Hospital, Parkville, VIC, Australia; 3 Department of Paediatrics, Melbourne University, Parkville, VIC, Australia; 4 Faculty of Health Sciences, Deakin University, Geelong, VIC, Australia; 5 Department of Pediatrics, University of Alberta, Edmonton, Canada; 6 Faculty of Medicine and Health Sciences, UCSI University, Kuala Lumpur, Malaysia; 7 Department of Pediatrics and Child Health, University of Manitoba, Winnipeg, Canada; 8 Department of Pediatrics, BC Children’s Hospital, University of British Columbia, Vancouver, Canada; 9 Department of Pediatrics, Hospital for Sick Children, University of Toronto, Toronto, Ontario, Canada; 10 Department of Medicine, McMaster University, Hamilton, Ontario, Canada; 11 Department of Physiology, University of Toronto, Toronto, Ontario, Canada; 12 Department of Anthropology, University of Toronto, Toronto, ON, Canada; 13 Division of Social and Behavioural Health Sciences, Dalla Lana School of Public Health, University of Toronto, Toronto, ON, Canada

**Keywords:** body composition, preclinical obesity, early childhood nutrition, milk fat intake, whole milk consumption, pediatrics, prospective cohort study

## Abstract

**Background:**

Guidelines in Canada, the United States, and other countries recommend that children switch from whole (3.25%) to reduced-fat milk after age 2 years to limit saturated fat intake and prevent obesity, despite these recommendations being derived primarily from adult studies. Emerging pediatric evidence challenges this approach, but prospective data in early school-aged children are scarce.

**Objectives:**

To test for cross-sectional and longitudinal associations of cow milk-fat content at age 5 years and adiposity indicators and obesity status among 5- and 8-year-old Canadian children.

**Methods:**

We analyzed data from the CHILD Cohort Study, a national longitudinal prospective pregnancy cohort study. At age 5 years, caregivers reported the fat content of cow milk consumed (skim [0%], 1%, 2%, and whole [3.25%]). Anthropometric measures at ages 5 years (*N* = 2043) and 8 years (*N* = 1574) included body mass index (BMI) and waist-to-height ratio *z*-scores; and at age 8 years, fat mass percentage (measured by bioelectric impedance analysis) and obesity (defined using World Health Organization criteria and the new clinical obesity Lancet Commission definitions). Multivariable linear and logistic regression models were adjusted for sociodemographic, lifestyle, and other dietary factors.

**Results:**

At age 5 years, most children consumed 2% (48.9%) or 3.25% (23.9%) fat from cow milk. Compared with skimmed cow milk, whole (3.25%) cow milk consumption was associated with lower BMI *z*-score at age 5 years [*β*: −0.34; 95% confidence interval (CI): −0.54, −0.13] and lower odds of living with obesity [odds ratio (OR): 0.22; 95% CI: 0.07, 0.67]. Whole cow milk consumption at 5 years was also inversely associated with BMI *z*-score (*β*: −0.42; 95% CI: −0.72, −0.11), waist-to-height ratio *z*-score (*β*: −0.35; 95% CI: −0.63, −0.07), fat mass (*β*: −1.58; 95% CI: −3.10, −0.06), obesity (OR: 0.31; 95% CI: 0.12, 0.80), and preclinical obesity (OR: 0.25; 95% CI: 0.09, 0.70) at age 8 years.

**Conclusions:**

Whole cow milk consumption at age 5 years was associated with lower adiposity and obesity indicators in middle childhood. These findings challenge current recommendations to limit milk fat in children and may inform future dietary guidelines.

## Introduction

In North America and many other countries, public health guidelines recommend that children transition from whole (3.25%) to reduced-fat cow milk after age 2 years [[1], [2], [3]] to limit saturated fat intake and reduce risk of obesity [[4], [5], [6]]. These guidelines are overwhelmingly based on extrapolation of data from adult studies and scant studies of children [[7], [8], [9], [10]]. Recent literature challenges the assumption that whole milk promotes excess adiposity in childhood; however, prospective studies incorporating robust body composition measures and contemporary definitions of obesity remain scarce [[11], [12], [13], [14]].

In 2019, Canada’s Food Guide was updated, no longer presenting “Milk and Alternatives” as a separate food group and removing age-specific serving recommendations [15]. Though there is no exclusive milk category or daily serving size recommendations, it recommends choosing lower-fat milk options as a beverage for all individuals older than 2 years. Nevertheless, milk remains a staple in early childhood diets, contributing substantially to energy intake: national surveillance data show that milk accounts for ≤15.6% of daily calories among Canadian children aged 2–5 years [16]. Understanding how variations in milk-fat content affect growth and adiposity in childhood is therefore critical to inform both policy and clinical recommendations.

Childhood obesity remains a pressing public health issue, with early excess adiposity often tracking into adulthood [17] and contributing to increased risk of cardiovascular disease, type 2 diabetes, and other chronic conditions [[18], [19], [20], [21]]. Although current guidelines emphasize reduced-fat milk, intervention and cohort studies in children and adolescents have not demonstrated adverse effects of whole milk on adiposity outcomes; in fact, several studies suggest an inverse association [[12], [13], [14],^22^]. A recent meta-analysis reported that children consuming whole-fat milk had 39% lower odds of overweight or obesity compared with those consuming reduced-fat milk, though study heterogeneity was high [23].

Despite this growing body of evidence, most longitudinal studies have focused on adolescence [13,24,25], leaving an important gap in understanding how milk-fat intake relates to adiposity in younger children, who consume milk in larger quantities [16]. To date, only 1 Canadian study has examined this association in early childhood, from 9 months to 8 years, using BMI *z*-scores and BMI-defined overweight/obesity as outcomes. Children who consumed whole milk were associated with lower BMI *z*-scores and lower odds of overweight/obesity compared with those consuming reduced-fat milk [22]. In the United States Project Viva cohort, compared with low-fat milk, higher-fat milk intake at age 3 years was also associated with lower odds of overweight/obesity but not with BMI *z*-scores in adolescence [13].

Other prospective studies examine milk intake in later childhood and adolescence. For example, in the Growing Up Today Study, children aged 9–14 years who consumed skim or 1% fat milk experienced greater BMI gain over time than those consuming higher-fat milk [26]. Findings from the Avon Longitudinal Study of Parents and Children (ALSPAC) in the United Kingdom show lower odds of overweight among 13-year-olds if they consumed full-fat dairy intake at the age of 10 years [25]. A study from Australia, the Raine study, did not find any associations between milk-fat intake and adiposity measures in adolescence [27]. Overall, results from prospective studies are inconsistent, and a few have evaluated milk-fat intake in school-aged children. Moreover, most studies have relied solely on BMI; although BMI is a widely used marker for obesity, it does not capture underlying body composition or fat distribution. The recent Lancet Diabetes & Endocrinology Commission advanced definitions of obesity beyond BMI, introducing preclinical and clinical classifications that better reflect the biological impact of excess adiposity [28].

The CHILD Cohort Study is one of the largest Canadian population-based pregnancy cohort studies originally established to examine the developmental origins of asthma and allergic diseases [29]. In addition to these primary outcomes, the CHILD study also examines other health outcomes, including growth, obesity, and cardiometabolic risk trajectories, across childhood [30]. Using data already collected, we aimed to examine both cross-sectional and longitudinal associations between cow milk-fat intake at age 5 years and adiposity and obesity indicators at ages 5 and 8 years. We hypothesized that children consuming cow milk with higher fat content (whole milk) compared with skim milk would have lower adiposity indicators and reduced odds of obesity across childhood.

## Methods

### Study population

The Canadian CHILD Cohort Study is a prospective population-based, pregnancy cohort study with participants from 4 sites across Canada (Vancouver, Edmonton, Manitoba, and Toronto) [29,30]. Females with singleton pregnancies enrolled between 2009 and 2012 were eligible if they delivered a healthy infant older than 34 weeks and 4 days of gestation, with a normal birth weight and no congenital abnormalities (*N =* 3454) [29]. In this study, we excluded participants who reported no cow milk consumption (7.3%) because the exposure of interest (milk-fat percentage) cannot be defined in the absence of milk intake. This approach is consistent with previous pediatric studies assessing milk-fat content [14,23]. Our study included participants with available data on milk-fat content at 5 years of age (*N* = 2124) and who had any adiposity and obesity outcomes at ages 5 years (*N =* 2043) and 8 years (*N =* 1574). The study visits occurred between 2013 to 2018 and 2018 to 2021, respectively. The participant flowchart is displayed in Supplementary Figure 1. This study was approved by the University of Toronto Ethics Board (RIS protocol number: 47810), and the CHILD study was approved by Human Research Ethics Boards at McMaster University, the Universities of Manitoba, Alberta, and British Columbia, and the Hospital for Sick Children. Informed consent was obtained from the caregivers.

### Assessment of milk-fat intake

As previously described [31,32], dietary intake at the 5-year visit was captured using a 114-item semiquantitative food frequency questionnaire (FFQ) completed by the caregiver. The FFQ was validated among a subset of the Family Atherosclerosis Monitoring in Early Life Study [33] for calcium and vitamin D [34]. In the FFQ, caregivers were asked “Do you give your child cow milk” with a yes/no option provided; and “how much fat does this milk contain” with the 5 options as “no fat (skim), 1% fat, 2% fat, 3.25% (homo), and >10% fat (cream).” For the analyses, we excluded participants who consumed >10% fat (cream) due to the small number in this category (*N* = 4). The FFQ also asked “How often has your child consumed cow milk in the past month?” for a volume of half cup/125 mL and frequency options given on a 9-level scale from “none to “>3/day.” Although the FFQ captured total cow milk consumption, it did not differentiate between plain and flavored (e.g., chocolate) milk; thus, any flavored cow milk was included in the overall milk category and classified by reported fat content.

Diet at the 8-year visit was assessed using the INTERHEART study food screener [35]. The question on dairy intake (includes milk, yogurt, cheese, curd, raita, lassi, custard, kloya, firni, kheer, milk puddings, and ice cream) was captured on a 4-point scale ranging from “Never,” “Monthly,” “Weekly,” and “Daily” and the number of times. Milk-fat intake was not captured at the 8-year visit [30,35].

### Assessment of adiposity indicators

At the 5- and 8-year visits, height was determined in a standing position to the nearest millimeter without shoes by a Harpenden Stadiometer, and weight was measured using a calibrated scale as previously reported [36]. Waist circumference (cm) was obtained using a nonstretchable measuring tape with an attached spring scale tension gauge (OHAUS Corp). We calculated age- and sex-adjusted BMI *z*-score using the WHO 2007 Child Growth Standards [37], which is recommended for Canadian health studies of participants who were 5–19 years old [38]. Waist-to-height ratio *z*-score was calculated based on the Third National Health and Nutrition Examination Survey Lambda–Mu–Sigma (LMS) table [39].

Fat mass percentage was measured using the Tanita Body Composition Analyzer (a bioelectrical impedance analyzer) at the 8-year visit only [30]. We classified children living with obesity as having a BMI *z*-score >2.0 using the WHO cutoffs [37]. In addition, at age 8 years, we classified children living with preclinical obesity and clinical obesity based on the recent Lancet Diabetes and Endocrinology Commission of Obesity [28].

In this new definition, obesity status incorporates confirmation of excess adiposity, such as using BMI and ≥1 other measurement of body size (e.g., waist circumference, waist-to-height), 2 measures of body size irrespective of BMI, or by body fat mass percentage alone. The definition also considers the presence of health complications, organ dysfunction, and/or compromised activities of daily living (ADL) to define preclinical and clinical obesity. Preclinical obesity refers to the presence of excess adiposity and no identified health complications due to obesity, signs or symptoms of organ dysfunction, or compromised ADL. Meanwhile, clinical obesity is further defined by evidence of health complications, organ dysfunction symptoms, and/or compromised ADL, such as elevated blood pressure or a cluster of unfavorable metabolic profiles (high levels of glucose, triglycerides, low density lipoprotein (LDL)-cholesterol, or low levels of high density lipoprotein (HDL)-cholesterol). Specifically in this study, to differentiate between clinical and preclinical obesity, the child's blood pressure (mmHg) and metabolic profile (glucose, triglyceride, and HDL/LDL levels) at 8 years of age were considered. In CHILD at the 8-year visit, blood pressure was measured using a validated automatic sphygmomanometer (Carescape Dinamap; GE Healthcare) in triplicate, with the average of the last 2 measurements used in analyses, and metabolic profile was obtained from high-throughput nuclear magnetic resonance spectroscopy (Nightingale Health Ltd) [40].

### Covariates

Maternal education level (completion of a postsecondary degree) was self-reported using questionnaires [29]. Previous studies have associated maternal education with children's dietary patterns [41] and obesity development [42]. Maternal BMI (kg/m^2^) was calculated using measured height and self-reported prepregnancy weight or estimated from weight measured 1 year postpartum if the prepregnancy weight was not recalled [43]. Maternal BMI is a strong predictor of childhood obesity [44]. Child weight at birth (kg) and biological sex (males vs. females) were obtained from medical records. These factors have been shown to impact the development of childhood obesity [45,46] and influence child dietary intake [47]. Child ethnicity was reported using the following questionnaire options: Caucasian White (referred to as White hereafter), Black, East Asian, First Nations, Hispanic, Middle Eastern, Mixed, Other, South Asian, and South-East Asian. For this analysis, children with parents from different ethnicities from one another (and not “other”) were categorized as “Multiracial,” and all non-White categories were grouped as "Other" [30]. Data on other confounders associated with obesity or child diet [[48], [49], [50]], such as exclusive breastfeeding at 6 months (yes vs. no), having older siblings (yes vs. no), study site (Edmonton, Manitoba, Toronto, and Vancouver), and organized physical activity (hours per week), were obtained via questionnaires.

Dietary covariates from FFQ at the 5-year visit included total daily energy intake (kcal/day), sugar-sweetened beverage (SSB) intake (cups/day) (orange juice, fruit drinks, regular and diet soda, and other juice), and total daily milk intake (cups/day). SSBs are hypothesized to displace dairy intake and are also strongly associated with the rise in overweight and obesity in children [51,52]. At the age of 8 years, from the food screener data [35], we derived the modified Alternative Healthy Eating Index (mAHEI) score [53] as described in detail previously [54].

### Statistical analysis

Participants’ descriptive characteristics were presented as mean (SD) for continuous variables with a normal distribution, median (IQR) for continuous variables without a normal distribution, and count (percentage) for categorical variables. To determine the cross-sectional and longitudinal association between cow milk-fat consumption and child adiposity outcomes, we ran multivariable-adjusted linear (for adiposity outcomes) and logistic (for obesity status) regression models, with skim milk as the reference category.

We examined a basic model adjusting for only energy (kcal/day) intake, whereas, in the multivariable-adjusted model, we further accounted for maternal prepregnancy BMI, maternal postsecondary education level, child’s ethnicity, child’s birth weight, breastfeeding exclusivity at 6 months of age, having older siblings, study site, and 5-year child characteristics SSB consumption, total milk volume intake, and physical activity. For the 8-year analysis, we additionally adjusted the model for diet quality (mAHEI score) and physical activity at the age of 8 years. Based on the sample sizes (*N* = 2043 at age 5 years; *N* = 1574 at age 8 years), the study had >80% power (*α*: 0.05) to detect a 0.25 SD difference in BMI *z*-score between milk and fat categories. For the smallest group contrast (skim milk *N* = 88 vs. whole milk *N* = 489), the minimal detectable difference at 80% power was 0.32 SD. Power may be limited for low-prevalence categorical outcomes such as clinical obesity.

### Sensitivity analyses

To determine if the estimated effects were independent of earlier BMI, we ran a sensitivity analysis by additionally adjusting the models for the change in BMI (from age 3 to 5 years) in the subset of children with available BMI data (*N* = 1960) for age 5-year analysis and (*N* = 1486) for age 8-year analysis. As a sensitivity analysis, models were further adjusted for total saturated fat intake (g/d) at age 5 years to assess whether associations were independent of overall dietary fat consumption. To consider the potential limitation of the low number of participants in the skim milk group, we repeated the analyses with the reference group changed to 1% fat milk. To determine if our longitudinal analyses were independent of concurrent dairy intake, we additionally accounted for dairy intake at 8 years. Furthermore, we repeated the analyses restricting to participants who had outcome data at both time points (*N* = 1545). We performed collinearity diagnostics and evaluated multicollinearity using the variance inflation factor (VIF), with all covariates showing VIF values below 1.5.

### Multiple imputation

To minimize potential bias from missing data (ranging between 0.12% and 14.9%), we imputed missing covariate values using the fully conditional specification method with predictive mean matching (*n* = 5 imputations), under the assumption of a nonmonotone missing data pattern [55]. We reported the pooled effect estimates from the multiple imputations. All analyses were conducted on SPSS, version 29.0 (IBM Corporation). Figures were created using the *ggplot2* package on R Studio, version 2024.12.1 (Posit Professional Business Continuity).

## Results

### Participant characteristics

**FIGURE 1:**
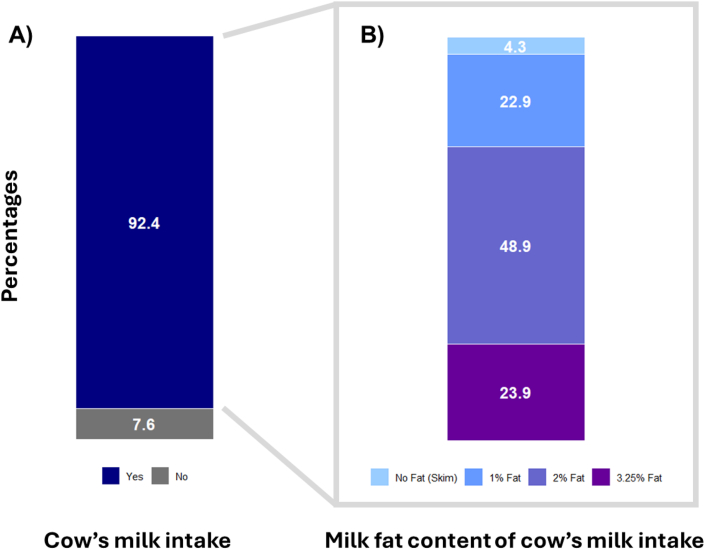
(A) Cow milk consumption in the CHILD Cohort Study, (B) proportion of each cow milk-fat content consumed at age 5 years in the CHILD Cohort Study (*N* = 2043). (A) Stacked bar graph of participants in the CHILD Cohort Study who consume cow milk at the age of 5 years; (B) stacked bar graph showing the proportion of each cow milk-fat content that is commonly consumed among participants who consume cow milk at the age of 5 years in the CHILD Cohort Study.

Table 1 shows the characteristics of the study participants at both time points; 5-year visit (*N* = 2043) and 8-year visit (*N* = 1574), respectively. The median age at the exposure assessment was 5.0 (IQR: 5.0–5.1) years. Approximately half of the children were male [1076 (52.7%); 834 (53.0%)], and the majority were of White ethnicity [1360 (66.6%) and 1069 (67.9%)] at the 5- and 8-year visits, respectively. In our cohort, 92.4% of children consumed cow milk at age 5 years (Figure 1A). Of them, 23.9% of children consumed 3.25% (whole) milk-fat content, with most children (48.9%) consuming 2% milk-fat content, whereas 22.8% consumed 1% milk-fat content and 4.3% consumed skim milk (Figure 1B).

**TABLE 1:** Participant characteristics in the CHILD Cohort Study

	5-y Analysis (*N* = 2043)	8-y Analysis (*N* = 1574)
Family characteristics		
Maternal postsecondary education (yes vs. no)	1610 (78.8)	1268 (80.6)
Maternal BMI (kg/m^2^)	24.8 (5.4)	24.8 (5.4)
Other siblings (yes vs. no)	969 (47.4)	759 (48.2)
Study site		
Edmonton	478 (23.4)	362 (23.0)
Manitoba	643 (31.5)	495 (31.4)
Toronto	447 (21.9)	273 (17.3)
Vancouver	475 (23.3)	444 (28.2)
Birth characteristics		
Child sex (males vs. females)	1076 (52.7)	834 (53.0)
Birth weight (kg)	3.5 (0.5)	3.5 (0.5)
Child ethnicity		
White	1360 (66.6)	1069 (67.9)
Multiracial	463 (22.7)	357 (22.7)
Other	220 (10.8)	148 (9.4)
Exclusive breastfeeding at 6 months (yes vs. no)	380 (18.6)	317 (20.1)
Childhood characteristics		
Daily caloric intake at age 5 years (kcal/d)	1503.2 (1235.8, 1813.5)	1498.0 (1236.0, 1804.0)
Volume of milk intake at age 5 years^1^		
Less than 1 cup a day	1155 (56.5)	890 (56.5)
One cup a day or more	888 (43.5)	684 (43.5)
Fat content of milk intake at age 5 years		
No fat (skim)	88 (4.3)	68 (4.3)
1% fat	466 (22.9)	372 (23.6)
2% fat	1000 (48.9)	739 (47.0)
3.25% fat	489 (23.9)	395 (25.1)
Age at assessment (y)	5.0 (5.0, 5.1)	8.4 (8.1, 8.8)
Physical activity (h/wk)	2.0 (1.0, 3.0)	7.0 (5.0, 11.4)
Modified Alternative Healthy Eating Index	NA	26.0 (7.4)
Daily dairy intake at age 8 years (yes vs. no)	NA	1244 (79)
Outcome characteristics		
BMI *z*-score	0.3 (0.9)	0.2 (1.2)
Waist-to-height *z*-score	−0.3 (1.0)	−0.5 (1.1)
Fat mass percentage (%)	NA	19.6 (6.0)
Obesity status^2^ (yes vs. no)	88 (4.3)	126 (8.0)
Preclinical obesity status^3^ (yes vs. no)	NA	83 (6.3)
Clinical obesity status^3^ (yes vs. no)	NA	48 (3.0)

Values are means ± SD for continuous variables with a normal distribution, numbers (%) for categorical variables, or medians (IQR) for continuous variables with skewed distribution based on nonimputed data.

1 One cup = 250 mL of cow milk per day.

2 Obesity status based on World Health Organization reference standards as BMI *z*-score > 2.00.

3 Based on the Lancet Commission definitions of obesity.

At age 5 years, 88 children (4.3%) were with obesity (based on the WHO cutoffs). By age 8 years, the number of children living with obesity (based on WHO cutoffs) was 126 (8.0%), whereas the number of children living with preclinical and clinical obesity (based on the Lancet Commission) was 83 (6.3%) and 48 (3.0%) children, respectively. Participant characteristics before and after imputation remained similar and are presented in Supplementary Table 1.

Compared with all participants included in the CHILD Cohort (*N* = 3545), participants included in this study had similar characteristics; however, participants included in this study had slightly lower total energy intake at age 5 years and a higher proportion of mothers with a postsecondary degree (Supplementary Table 2). Supplementary Table 3 shows study participant characteristics across milk-fat intake categories at age 5 years. Maternal prepregnancy BMI was lower among children who consumed 3.25% fat milk; however, there were no statistical differences in birth weight across all milk-fat intake categories. Interestingly, most children consuming skim milk and 1% fat milk had older siblings. Additionally, children of White ethnicity tend to consume cow milk with 1% fat over the other milk-fat levels, whereas those with a Multiracial ethnic background tend to consume whole (3.25%) milk.

### Association between milk-fat percentage intake and adiposity indicators and obesity status at age 5 years

**FIGURE 2:**
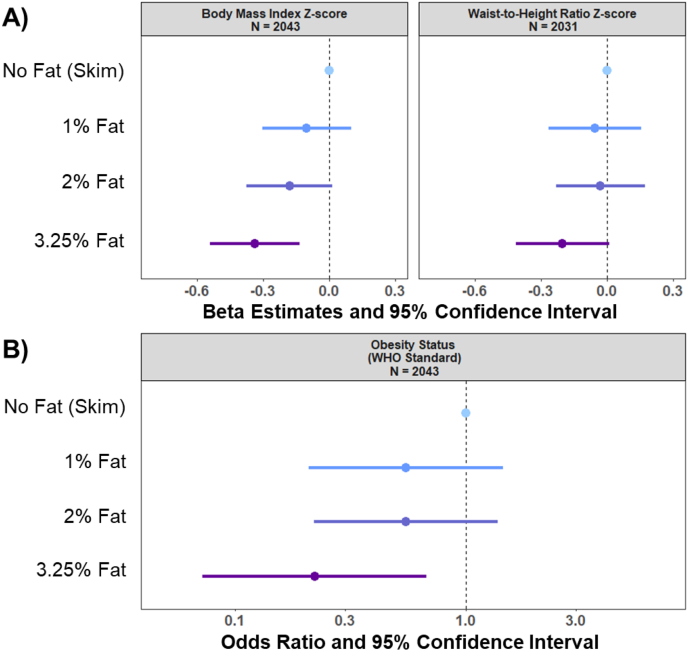
Cross-sectional associations of milk-fat content, adiposity indicators, and obesity status at age 5 years in the CHILD Cohort Study (*N* = 2043). Values are as follows: (A) β-estimates representing standardized mean differences with 95% confidence interval from linear regression analyses for BMI *z*-score and waist-to-height *z*-score, for children in a given milk-fat category compared with the reference group (skim milk) at age 5 years; (B) odds ratios and 95% confidence interval from logistic regression analysis of milk-fat content at age 5 years and obesity status at age 5 years. The multivariable-adjusted analyses account for maternal BMI, maternal postsecondary education level, child’s race, child’s birth weight, breastfeeding exclusivity, having older siblings, energy intake at 5 years of age, sugar-sweetened beverage consumption at 5 years of age, milk consumption at 5 years of age, organized physical activity at age 5 years of age, and study center site. WHO, World Health Organization. Number of participants in each group per analysis is as follows: BMI *z*-score: no fat (skim) *n* = 88, 1% fat *n* = 466, 2% fat *n* = 1000, 3.25% fat *n* = 489; waist-to-height ratio *z*-score: no fat (skim) *n* = 88, 1% fat *n* = 464, 2% fat *n* = 992, 3.25% fat *n* = 487; obesity status: no fat (skim) *n* = 88, 1% fat *n* = 466, 2% fat *n* = 1000, 3.25% fat *n* = 489.

Figure 2A shows the multivariable-adjusted linear regression models on the cross-sectional associations of milk-fat content at age 5 and BMI *z*-score and waist-to-height ratio *z*-score. The results are beta estimate (standardized mean difference) and 95% confidence interval (95% CI) from linear regression models for each adiposity indicator (BMI *z*-score, waist-to-height ratio *z*-score) for children in a given milk-fat group compared with the reference group (children who consume skim milk). We found that children consuming 3.25% milk fat had a lower BMI *z*-score compared with those consuming skim milk (*β*: −0.34; 95% CI: −0.54, −0.13). Although the other milk-fat groups were not statistically significant, we did observe a dose–response trend for BMI *z*-score with increasing fat content in cow milk. No significant association was observed between milk-fat content and waist-to-height ratio *z*-score (*β*: −0.20; 95% CI: −0.41, 0.01).

In logistic regression models, where the values represent the odds ratio (OR) and 95% CI on the outcome (obesity), children who consumed 3.25% milk fat had 78% lower odds of living with obesity (OR: 0.22; 95% CI: 0.07, 0.67; *P*: 0.01) compared with those consuming skim milk (reference group) (Figure 2B). These findings were similar in the basic adjusted models that accounted only for energy intake (Supplementary Figure 2). Furthermore, the associations of milk fat with BMI *z*-score remained statistically significant after adjusting for change in BMI from age 3 to 5 years, although the effect estimates slightly attenuated (*β*: −0.26; 95% CI: −0.44, −0.08) (Supplementary Figure 3A). The associations with obesity were no longer significant when accounting for the change in BMI from 3 to 5 years (OR: 0.36; 95% CI: 0.09, 1.41; *P*: 0.14) (Supplementary Figure 3B). When additionally adjusting for total saturated fat intake, the magnitude and direction of associations remained unchanged, indicating that observed effects were not explained by differences in overall dietary fat intake (Supplementary Figure 4). Compared with the main analyses, we observed similar results when we changed the reference group to 1% milk fat (Supplementary Figure 5). Restricting the analyses to participants with outcome data at both time points (*N* = 1545) showed similar results to the main analyses (Supplementary Figure 6).

### Association between milk-fat percentage intake at age 5 y and adiposity indicators and obesity status at age 8 years

**FIGURE 3:**
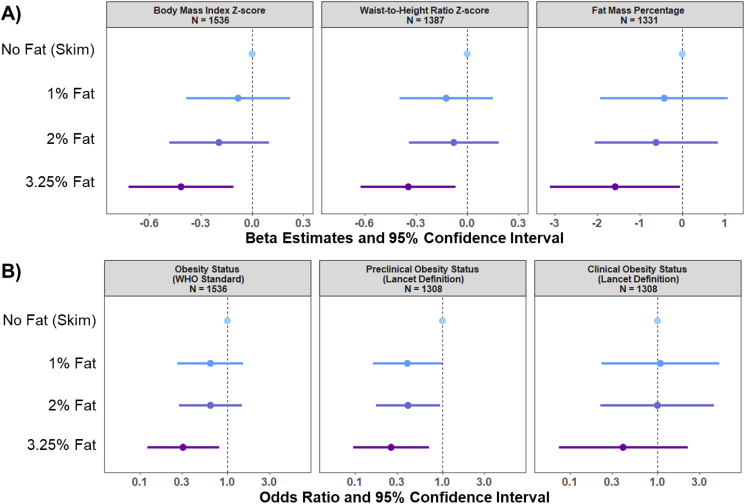
Associations of milk-fat content at age 5 years, adiposity indicators, and obesity status at age 8 years in the CHILD Cohort Study (*N* = 1574). Values are as follows: (A) β estimates representing standardized mean differences with 95% confidence interval from linear regression analyses for children in a given milk-fat category compared with the reference group (skim milk) at age 5 years for BMI z-score and waist-to-height *z*-score, and the mean difference in fat mass percentage at age 8 years; (B) odds ratios and 95% confidence interval from logistic regression analysis of milk-fat content at age 5 years and obesity status at age 8 years. The multivariable-adjusted analyses account for maternal BMI, maternal postsecondary education level, child’s race, child’s birth weight, breastfeeding exclusivity, having older siblings, energy intake at 5 years of age, sugar-sweetened beverage consumption at 5 years of age, milk consumption at 5 years of age, modified Alternative Healthy Eating Index (mAHEI) score, organized physical activity at age 8 years of age, and study center site. The model for fat mass percentage additionally accounted for age and sex. WHO = World Health Organization. Number of participants in each group per analysis is as follows: BMI *z*-score: no fat (skim) *n* = 67, 1% fat *n* = 365, 2% fat *n* = 714, 3.25% fat *n* = 390; waist-to-height ratio *z*-score: no fat (skim) *n* = 64, 1% fat *n* = 332, 2% fat *n* = 646, 3.25% fat *n* = 345; Fat mass percent: no fat (skim) *n* = 63, 1% fat *n* = 321, 2% fat *n* = 613, 3.25% fat *n* = 334; Obesity status: no fat (skim) *n* = 67, 1% fat *n* = 365, 2% fat *n* = 714, 3.25% fat *n* = 390; preclinical obesity status: no fat (skim) *n* = 60, 1% fat *n* = 315, 2% fat *n* = 609, 3.25% fat *n* = 324; clinical obesity status: no fat (skim) *n* = 60, 1% fat *n* = 315, 2% fat *n* = 609, 3.25% fat *n* = 324.

Intake of milk with higher fat content at age 5 years was inversely associated with adiposity and obesity indicators at age 8 years (Figure 3). Particularly, children who consumed 3.25% milk fat had significantly lower BMI *z*-score (*β*: −0.42; 95% CI: −0.72, −0.11), waist-to-height ratio *z*-score (*β*: −0.35; 95% CI: −0.63, −0.07), and fat mass percentage (*β*: −1.58; 95% CI: −3.10, −0.06) compared with those who consumed skim milk (Figure 3A).

Logistic regression analyses showed that compared with children consuming skim milk, children consuming 3.25% milk had 69% lower odds of living with obesity (OR: 0.31; 95% CI: 0.12, 0.80; *P*: 0.02) and 75% lower odds of having preclinical obesity (OR: 0.25; 95% CI: 0.09, 0.70; *P*: 0.01) at age 8 years (Figure 3B). Preclinical obesity ORs were also lower among children consuming 1% or 2% milk fat compared with children consuming skim milk, although borderline significant (Figure 3B). No significant associations were observed between milk-fat content and clinical obesity. All results were similar in the basic adjusted models, accounting only for energy intake (Supplementary Figure 7). These findings were consistent in sensitivity analyses adjusting for changes in BMI in early childhood (Supplementary Figure 8), adjustment for total saturated fat intake at 5 years (Supplementary Figure 9), and adjustment for total dairy intake at age 8 years (Supplementary Figure 10). Most of our findings remain consistent when we use 1% milk fat as the reference category (Supplementary Figure 11). When restricting these analyses among participants with data at both time points (*N* = 1545), the results were consistent with those of the main analyses (Supplementary Figure 12).

## Discussion

To the best of our knowledge, this is the first Canadian study examining both cross-sectional and prospective associations between cow milk-fat intake and multiple adiposity indicators and preclinical and clinical obesity in childhood. Our findings showed that consumption of whole milk-fat content at age 5 years was consistently associated with lower adiposity measures, including BMI *z*-score, waist-to-height ratio *z*-score, and fat mass percentage, as well as lower odds of living with obesity at ages 5 and 8 years. Notably, our study is also among the first to apply the Lancet Commission framework to define preclinical and clinical obesity status in children, offering a more nuanced understanding of early obesity risk. Although associations with clinical obesity were not statistically significant, this likely reflects the low number of children meeting the stringent Lancet Commission definition, which requires evidence of metabolic or organ dysfunction in addition to excess adiposity. At ages 5–8 years, most children with elevated adiposity have not yet developed metabolic complications, which may explain the absence of detectable group differences for clinical obesity. Nevertheless, the consistent inverse associations observed for continuous adiposity measures, including BMI *z*-score, waist-to-height ratio, and fat mass percentage, suggest that differences in adiposity emerge well before clinical thresholds are reached. These subclinical differences may be of clinical relevance, as even small reductions in BMI *z*-score (≥0.1) have been associated with improved cardiometabolic markers in children [56].

Almost half of the children in our study (56.5%) consumed <1 cup of milk per day, which was below the 2007 Canada Food Guide recommendations for children aged 4 to 8 years, which advised 2–3 servings of milk and alternatives (including other dairy and nondairy products) daily [57]. Overall milk intake in the CHILD Cohort was modest compared with other pediatric cohorts, including Target Kids! (∼1.9 cups/day) [22] and Project Viva (∼2.3 times/day) [13]. Despite this relatively low consumption, we observed consistent inverse associations between milk-fat content and adiposity indicators. Similar associations have been reported in studies of children with higher milk intakes, suggesting that the composition of milk fat, rather than the total quantity consumed, may be more relevant to adiposity and obesity risk in childhood. When examining the type of milk consumed, nearly half of the children (48.9%) consumed 2% milk, consistent with recommendations to transition from whole to reduced-fat milk after age 2 years [4,5], which is similar to other studies [58].

The results from our study are consistent with recent studies assessing cow milk-fat intake and adiposity outcomes in young children [13,22]. A meta-analysis conducted a subgroup analysis of 8 cross-sectional and prospective cohort studies of children aged 1–5 years using 8 cross-sectional and prospective cohort studies and found that children who consumed whole milk had lower odds of living with overweight or obesity (OR, 0.61) compared with those consuming reduced-fat milk [23]. Similarly, our findings align with previous prospective studies, showing that associations between milk-fat and adiposity outcomes have been similar to our findings. For example, the Canadian TARGet Kids! prospective study found that among 7467 children, each 1% increase in cow milk-fat content was associated with 0.05 lower BMI *z*-score across childhood (aged 9 months to 8 years) [22]. Unlike our study, which included multiple adiposity outcomes, Target Kids! examined only BMI and BMI-defined overweight/obesity.

Our findings also align with the United States Project Viva, which assessed milk-fat intake at the age of 3 years and adiposity outcomes at the age of 13 years among 796 children, and found 40% lower odds of overweight or obesity for higher-fat milk (whole or 2%) compared with lower-fat milk (1% or skim) [13]. This study grouped whole milk and 2% as higher-fat milk, and 1% and skim as lower-fat milk, whereas our study looked at each individual group separately. Additionally, they had a longer follow-up duration between their exposure and outcome assessment. In contrast, the short-term randomized trial, the Milky Way Study, of 5-year-old children found no significant differences in BMI *z*-scores between those consuming full-fat versus low-fat dairy; however, the trial was limited by its short duration (3 months) and small study population size (*N* = 49) [12].

Inconsistent findings are more evident in studies of older children and adolescents, and in those assessing total dairy intake rather than milk-fat percentage. Most prior work has relied primarily on BMI as the sole indicator of adiposity. In the United Kingdom ALSPAC study, adolescents in the highest quartile of full-fat dairy intake had lower odds of being overweight [25]. However, in their analyses, the authors aggregated all dairy products [white milk (cow, sheep, and goat), flavored milk, cheese, yogurt, and ice cream], making it difficult to isolate the effects of cow milk. Similarly, the Australian Raine Study compared total regular-fat versus reduced-fat dairy and found no significant associations after accounting for confounding factors (e.g., breastfeeding and fitness) [27].

Our study focused specifically on cow milk-fat percentage and extended beyond BMI to include waist-to-height ratio and fat mass percentage, both stronger indicators of central and total adiposity. We further incorporated these measures into definitions of preclinical and clinical obesity based on the Lancet Commission framework, providing a more granular assessment of early obesity risk than prior work. Variations across studies likely reflect differences in exposure definition (milk fat vs. total dairy), age at exposure, outcome measures, and length of follow-up. All these factors could potentially contribute to the discrepancies among findings in observational studies; therefore, data should be interpreted carefully, and future large clinical trials are necessary.

Several biological pathways may explain the inverse associations we observed between milk-fat intake and adiposity outcomes in childhood. Cow milk fat contains bioactive compounds that may enhance satiety through hormonal pathways involving cholecystokinin and glucagon-like peptide-1 [59,60], potentially reducing overall caloric intake or displacing nutrient-poor foods. However, in our analysis, we accounted for total energy intake, other diet components, including SSB consumption and overall dietary quality, and then further adjusted for total saturated fat intake in a sensitivity analysis, and still observed consistent results.

Another potential pathway involves the “dairy matrix,” in which the combination of micronutrients, macronutrients, and the physical structure of dairy products produces metabolic effects beyond those of individual components [[61], [62], [63]]. Although protein and calcium content are consistent across milk-fat levels, differences in lipid composition, physical structure, and nutrient interactions may uniquely influence adiposity regulation. Furthermore, the compositional difference between 2% and skim milk is smaller than that between whole (3.25%) and skim milk, potentially insufficient to elicit measurable metabolic or satiety effects. This might explain why associations were more evident for whole milk than for 2% milk. Together, these potential mechanisms suggest that milk fat may influence satiety, energy balance, and body composition through multiple interacting biological pathways.

The selection of milk-fat content by parents based on a child’s body size could also be a contributing mechanism. Parents of children with higher adiposity indicators may preferentially purchase lower-fat milk for child consumption. This behavior could potentially introduce reverse causation, as the observed association between milk-fat content and adiposity may partly reflect the child’s pre-existing adiposity influence on milk-fat selection and consumption, rather than a causal effect of milk-fat consumption on child adiposity. In the 5-year analysis, when we accounted for the BMI change in early childhood, the effect estimates were slightly attenuated, although they remained statistically significant, which might be explained by potential reverse causality. Further mechanistic and longitudinal studies are needed to clarify the role of specific milk-fat constituents in early life adiposity and obesity development.

Our findings add to a growing body of evidence, even within a cohort characterized by modest cow milk consumption. This suggests that the relationship may not simply reflect total milk quantity but rather the composition of milk fat itself. Moreover, in populations with higher obesity prevalence or greater milk intake, these associations could be more pronounced. Thus, our findings highlight that even small differences in milk-fat exposure during early childhood may carry measurable implications for adiposity trajectories. These results hold important implications for population-level dietary guidance and early prevention strategies, particularly in the context of rising childhood obesity. Current guidelines recommend transitioning to reduced-fat milk after age 2 years to reduce saturated fat intake; however, our results challenge this assumption. Even modest reductions in BMI *z*-score, as little as 0.1, have been associated with improvements in cardiometabolic markers, including insulin, total cholesterol, and LDL-cholesterol [56]. Thus, the estimated effect sizes observed in our study (−0.34 and −0.42 lower BMI *z*-scores at ages 5 and 8 years, respectively) represent clinically meaningful differences. Although further randomized trials and mechanistic studies are needed to establish causality and identify optimal dairy fat recommendations in early childhood, our results suggest that current guidelines may not align with emerging evidence. Revisiting these recommendations could have important implications for early life nutritional counseling, clinical practice, and national food policy.

This study has several notable strengths. To our knowledge, it is the first national, longitudinal Canadian study to examine cow milk-fat intake in relation to a broad set of adiposity indicators and obesity status in young children, both cross-sectionally and prospectively [30]. We incorporated multiple markers of adiposity, including BMI *z*-score, waist-to-height ratio, fat mass percentage, and standardized obesity definitions from both the WHO and the Lancet Commission, which allowed a more nuanced and potentially clinically relevant assessment of early obesity risk [28]. These approaches enhance the clinical relevance of our findings by capturing early metabolic risk and by framing obesity as a complex, chronic condition rather than solely a weight-based classification. To assess the impact of unequal group sizes, analyses were repeated using 1% milk as the reference group and in a subset with complete data at both time points (*N* = 1545). Results remained consistent, suggesting minimal bias from unequal sample sizes.

Beyond its national importance, this work contributes to the global evidence base on dairy consumption and child health. A few large, prospective studies internationally have examined milk-fat intake in early childhood with obesity and adiposity outcomes. By leveraging data from a large, well-characterized pregnancy cohort and by conducting extensive sensitivity analyses, our study adds methodological rigor and strengthens confidence in the findings. Collectively, these strengths position our study to inform not only Canadian dietary guidance but also broader international discussions on the role of dairy fat in early life nutrition and obesity prevention.

Our study also has limitations. There may be potential recall bias in food intake frequency at age 5 years, an inherent limitation of self-reported FFQs. Although flavored milk was not separately reported in the FFQ, such products in Canada are typically 1% or 2% milk and represent <8% of total milk consumption in children [64]; thus, misclassification across milk-fat categories would be minimal and nondifferential. We did not capture milk-fat intake or daily cow milk volume at age 8 years, which restricts interpretation of concurrent intake at the outcome assessment and limits insight into changes in milk consumption between ages 5 and 8 years. Additionally, although the 8-year dietary screener did list examples of dairy items, there may be subjective interpretations on what constitutes “dairy,” potentially resulting in recall or classification bias.

The generalizability of these findings should be interpreted in the context of the CHILD Cohort. As a multicenter, population-based study recruiting families from 4 Canadian provinces, CHILD reflects diverse geographic and environmental settings. Enrolled families were more likely to have higher socioeconomic status and educational attainment than the broader Canadian population, which may limit the generalizability of findings to more socioeconomically or ethnically diverse groups. Participants included in our analyses were more likely to be of White ethnicity and have mothers with higher education compared with the overall CHILD Cohort, which may introduce selection bias and limit the generalizability of our findings to more diverse populations. As well, milk consumption patterns and public health guidance differ across countries; therefore, our findings are most generalizable to populations with similar dietary practices and food environments, such as Canada and the United States. Replication in other settings, including low- and middle-income contexts or those with differing dairy intakes, would strengthen external validity. In addition, parents may have selected the fat content of cow milk based on their child’s body size, raising the possibility of reverse causality; however, the CHILD Cohort did not collect data on reasons for milk choice. Last, despite adjustment for many potential confounders, due to the observational nature of our study, residual confounding cannot be ruled out.

In conclusion, in this national prospective cohort study, consumption of whole (3.25%) cow milk at age 5 years was consistently associated with lower adiposity indicators and reduced odds of obesity and preclinical obesity across childhood, when compared with children who consume cow milk without any milk fat (skim). These findings contribute to the accumulating evidence that challenges current public health recommendations favoring reduced-fat milk for children over age 2 years and underscore the need to re-evaluate dietary guidelines. Further research, including randomized trials and mechanistic studies, is warranted to determine whether current guidelines should be re-evaluated in light of emerging evidence.

## Author contributions

The authors’ responsibilities were as follows – KM: designed and managed this project and obtained funding; PS, TJM, ES, PJM, SET (The CHILD Study Founding team): conceived the CHILD Cohort design, managed study recruitment and oversaw clinical assessments of study participants; DWS: provided feedback on the analyses. DL, DB, PH, GM, TM, ZHC: supported deriving the Lancet Commission obesity data; TZ, ZHC: conducted all the statistical analyses and have primary responsibility for the final content; TZ, ZHC, KM: interpreted the data and wrote the manuscript; and all authors: read and approved the final manuscript.

## Data availability

Data described in the manuscript and analytic code will be made available upon request pending approval from CHILD's Access and Publication Committee and the CHILD Study National Coordinating Centre. A list of variables available in the CHILD Cohort Study is available at https://childstudy.ca/for-researchers/study-data/. Researchers interested in collaborating on a project and accessing CHILD Cohort Study data should contact the Study’s National Coordinating Centre (NCC) to discuss their needs before initiating a formal request. To contact the NCC, please email child@mcmaster.ca. More information about data access for the CHILD Cohort Study can be found at https://childstudy.ca/for-researchers/data-access/.

## Funding

The CHILD Cohort Study was funded by the Canadian Institutes of Health Research (CIHR) and the Allergy, Genes and Environment (AllerGen) Network of Centres of Excellence (NCE). GENOME CANADA also provided core funding for CHILD. These entities had no role in the design and conduct of the study; collection, management, analysis, and interpretation of the data; preparation, review, or approval of the manuscript; or decision to submit the manuscript for publication. This work was funded by CIHR Operating Grant and the Joannah & Brian Lawson Centre for Child Nutrition (made possible through a donation by President’s Choice Children’s Charity). DB is supported by National Health and Medical Research Council (NHMRC) Australia Investigator Grant. PJM is supported by the Women’s and Children’s Health Research Institute. TJM is supported by CIHR, Cystic Fibrosis Canada and the Physician’s Services Incorporated Foundation. KM is supported by CIHR and Heart & Stroke Foundation. Funders have no role in project management, design, sampling, analysis, interpretation or writing of this article.

## Conflict of interest

The authors report no conflicts of interest.
