# Supplementary material for: Milk fat intake, adiposity, and obesity in Canadian children: findings from the prospective Canadian CHILD Cohort Study
Source: Am J Clin Nutr. 2026 Apr 7;123(5):101186. doi: 10.1016/j.ajcnut.2025.101186 (PMC13197898; doi:10.1016/j.ajcnut.2025.101186)
Supplement: Multimedia component 1 [file mmc1.docx]

# **SUPPLEMENTARY MATERIAL**

**Milk Fat Intake, Adiposity and Obesity in Canadian Children: Findings from the Prospective Canadian CHILD Cohort Study**

List of Authors: Tara Zeitoun^1†^, Zheng Hao Chen^1†^, David Burgner^2,3,4^, Gabbi MacKechnie^2^, Prue Huntington^2^, Toby Mansell^2,3^, Danielle Longmore^2,4^, Piushkumar J. Mandhane^5,6^, Elinor Simons^7^, Stuart E. Turvey^8^, Padmaja Subbarao^9,10,11^, Theo J. Moraes^9^, Daniel W Sellen ^1, 13, 14^, Kozeta Miliku ^1, 9^*

| **Supplementary Figure 1** | Flowchart of the Study Participants Included in the Analysis |
| --- | --- |
| **Supplementary Table 1** | Descriptive characteristics of the original and multiple imputed datasets (N = 2043) in the CHILD Cohort Study |
| **Supplementary Table 2** | Descriptive characteristics of participants enrolled in the study (N = 3454), those with five-year outcome data (N = 2043), and those with eight-year outcome data (N = 1574): the CHILD Cohort Study |
| **Supplementary Table 3** | Descriptive characteristics of participants enrolled in the study (N = 2043) stratified across milk fat content at age five |
| **Supplementary Figure 2** | Associations of milk fat content at age five, adiposity indicators and obesity status at age five from basic adjusted analyses (N = 2043) |
| **Supplementary Figure 3** | Associations of milk fat content at age five, adiposity indicators and obesity status at age five, additionally accounting for change in BMI from ages three to five (N = 2043) |
| **Supplementary Figure 4** | Associations of milk fat content at age five, adiposity indicators and obesity status at age five, additionally accounting for total saturated fat intake at age three (N= 2043) |
| **Supplementary Figure 5** | Associations of milk fat content at age five, adiposity indicators and obesity status at age five, with 1% milk fat as the reference (N = 2043) |
| **Supplementary Figure 6** | Associations of milk fat content at age five and adiposity indicators at age five, restricted among participants with obesity data at both time points (N = 1545) |
| **Supplementary Figure 7** | Associations of milk fat content at age five, adiposity indicators and obesity status at age eight from basic adjusted analyses (N = 1574) |
| **Supplementary Figure 8** | Associations of milk fat content at age five, adiposity indicators and obesity status at age eight, additionally accounting for change in BMI from ages three to five (N = 1574) |
| **Supplementary Figure 9** | Associations of milk fat content at age five, adiposity indicators and obesity status at age five, additionally accounting for total saturated fat intake at age three (N= 1574) |
| **Supplementary Figure 10** | Associations of milk fat content at age five, adiposity indicators and obesity status at age eight, accounting for dairy intake at age eight (N = 1574) |
| **Supplementary Figure 11** | Associations of milk fat content at age five, adiposity indicators and obesity status at age eight, with 1% milk fat as the reference (N = 1574) |
| **Supplementary Figure 12** | Associations of milk fat content age five and adiposity indicators at age eight, restricted among participants with obesity data at both time points (N = 1545) |

## **Supplementary Figure 1: Flowchart of the Study Participants Included in the Analysis**

Participants with milk fat content data

**N = 2124**

FFQ data available at 5 years of age

**N = 2305**

Participants eligible at birth

**N = 3454**

**N = 1149** e**xcluded due to:**

Withdrawal (N = 234)

No FFQ data available at 5 years of age (N = 915)

**N = 181** e**xcluded due to:**

No consumption of cow's milk (N = 177)

Consuming milk fat content of >10% fat (N = 4)

Pregnant women recruited in the CHILD study

**N = 3621**

**N = 167 excluded due to:**

Withdrawal (N = 8)

Ineligibility (miscarriage, preterm birth,
or congenital abnormalities) (N = 159)

Children with milk fat data at 5-years and at least one obesity outcome at 8-years

**N = 1574**

Body mass index z-score:

Waist to height ratio z-score:

Fat mass percentage:

Obesity status
(WHO standard):

Preclinical obesity status
(Lancet definition):

Clinical obesity status
(Lancet definition):

**N = 550** e**xcluded due to:**

Loss to follow-up at 8-year visit (N = 244)

No obesity outcome at 8-year visit (N = 306)

Children with milk fat data at 5-years and at least one obesity outcome at 5-years

**N = 2043**

N = 1536

N = 1387

N = 1331

N = 1536

N = 1308

N = 1308

Body mass index z-score:

Waist to height ratio z-score:

Obesity status
(WHO standards):

N = 2043

N = 2031

N = 2043

**N = 81** e**xcluded due to:**

No obesity outcome at 5-year visit

## **Supplementary Table 1. Descriptive characteristics of the original and multiple imputed datasets (N= 2043) in the CHILD Cohort Study**

|  | **Original  (N = 2043)** | **Imputed**  **(N = 2043)** |
| --- | --- | --- |
|  |  |  |
| Family Characteristics |  |  |
| Maternal Post-Secondary Education (Yes vs. No) | 1586 (77.6) | 1610 (78.8) |
| Missing | 39 (1.9) | 0 (0.0) |
| Maternal BMI (kg/m²) | 24.8 (5.4) | 24.8 (5.4) |
| Missing | 57 (2.8) | 0 (0.0) |
| Other Siblings (Yes vs. No) | 969 (47.4) | 969 (47.4) |
| Missing | - | - |
| Study Site |  |  |
| Edmonton | 478 (23.4) | 478 (23.4) |
| Manitoba | 643 (31.5) | 643 (31.5) |
| Toronto | 447 (21.9) | 447 (21.9) |
| Vancouver | 475 (23.3) | 475 (23.3) |
| Missing | - | - |
| Birth Characteristics |  |  |
| Child Sex (Males vs. Females) | 1076 (52.7) | 1076 (52.7) |
| Missing | - | 0 (0.0) |
| Birth Weight (kg) | 3.5 (0.5) | 3.5 (0.5) |
| Missing | 42 (2.1) | 0 (0.0) |
| Child Ethnicity |  |  |
| Caucasian White | 1354 (66.3) | 1360 (66.6) |
| Multiracial | 460 (22.5) | 463 (22.7) |
| Other | 220 (10.8) | 220 (10.8) |
| Missing | 9 (0.4) | 0 (0.0) |
| Exclusive Breastfeeding at 6 Months (Yes vs. No) | 378 (18.5) | 380 (18.6) |
| Missing | 28 (1.4) | 0 (0.0) |
| Childhood Characteristics |  |  |
| Daily Caloric Intake (kcal/day) | 1503.2 [1235.8, 1813.5] | 1503.2 [1235.8, 1813.5] |
| Missing | - | - |
| Volume of Milk Intake (cup/day) | 0.5 [0.4, 1.3] | 0.5 [0.4, 1.3] |
| Missing | - | - |
| Milk Fat Content |  |  |
| No Fat (Skim) | 88 (4.3) | 88 (4.3) |
| 1% Fat | 466 (22.8) | 466 (22.8) |
| 2% Fat | 1000 (48.9) | 1000 (48.9) |
| 3.25% Fat | 489 (23.9) | 489 (23.9) |
| Missing | - | - |
| Exact Age at Outcome Assessment (years) | 5.0 [5.0, 5.1] | 5.0 [5.0, 5.1] |
| Missing | - | - |
| Physical Activity (hours/week) | 2.0 [1.0, 3.0] | 2.0 [1.0, 3.0] |
| Missing | 121 (5.9) | 0 (0.0) |

Values are means ± SD for continuous variables with a normal distribution, numbers (%) for categorical variables, or medians [IQR] for continuous variables with a skewed distribution. BMI = body mass index.

## **Supplementary Table 2. Descriptive characteristics of participants enrolled in the study (N= 3454), those with five-year outcome data (N= 2043), and those with eight-year outcome data (N= 1574): the CHILD Cohort Study**

|  | **Participants enrolled in the study  (N= 3454)** | **Participants with five-year outcomes (N= 2043)** | **Participants with eight-year outcomes (N= 1574)** |
| --- | --- | --- | --- |
| **Family Characteristics** | | | |
| Maternal Post-Secondary Education (Yes vs. No) | 2389 (69.2) | 1586 (77.6) | 1246 (79.2) |
| Missing | 327 (9.5) | 39 (1.9) | 32 (2.0) |
| Maternal BMI (kg/m²) | 24.8 (5.5) | 24.8 (5.4) | 24.7 (5.3) |
| Missing | 518 (15.0) | 57 (2.8) | 44 (2.8) |
| Other Siblings (Yes vs. No) | 1512 (43.8) | 969 (47.4) | 759 (48.2) |
| Missing | 192 (5.6) | 0 (0.0) | 0 (0.0) |
| Study Site |  |  |  |
| Edmonton | 811 (23.5) | 478 (23.4) | 362 (23.0) |
| Manitoba | 1043 (30.2) | 643 (31.5) | 495 (31.4) |
| Toronto | 813 (23.5) | 447 (21.9) | 273 (17.3) |
| Vancouver | 787 (22.8) | 475 (23.3) | 444 (28.2) |
| Missing | 0 (0.0) | 0 (0.0) | 0 (0.0) |
| **Birth Characteristics** | | | |
| Child Sex (Males vs. Females) | 1816 (52.6) | 1076 (52.7) | 834 (53.0) |
| Missing | 0 (0.0) | 0 (0.0) | 0 (0.0) |
| Birth Weight (kg) | 3.5 (0.05) | 3.5 (0.5) | 3.5 (0.5) |
| Missing | 260 (7.5) | 42 (2.1) | 35 (2.2) |
| Child Ethnicity |  |  |  |
| White | 2046 (59.2) | 1354 (66.3) | 1064 (67.6) |
| Multiracial | 745 (21.6) | 460 (22.5) | 355 (22.6) |
| Other | 420 (12.2) | 220 (10.8) | 148 (9.4) |
| Missing | 243 (7.0) | 9 (0.4) | 7 (0.4) |
| Exclusive Breastfeeding at 6 Months (Yes vs. No) | 552 (16.0) | 378 (18.5) | 316 (20.1) |
| Missing | 412 (11.9) | 28 (1.4) | 18 (1.1) |
| **Childhood Characteristics** | | | |
| Daily Caloric Intake at Age Five (kcal/day) | 1589.1  [1227.0, 1810.6] | 1503.2  [1235.8, 1813.5] | 1498.0  [1236.0, 1804.0] |
| Missing | 1154 (33.4) | 0 (0.0) | 0 (0.0) |
| Volume of Milk Intake (cup/day) | 0.5 [0.4, 1.3] | 0.5 [0.4, 1.3] | 0.5 [0.4, 1.3] |
| Missing | 1326 (38.4) | 0 (0.0) | 0 (0.0) |
| Milk Fat Content |  |  |  |
| No Fat (Skim) | 95 (2.8) | 88 (4.3) | 68 (4.3) |
| 1% Fat | 478 (13.8) | 466 (22.8) | 372 (23.6) |
| 2% Fat | 1037 (30.0) | 1000 (48.9) | 739 (47.0) |
| 3.25% Fat | 514 (14.9) | 489 (23.9) | 395 (25.1) |
| Missing | 2124 (61.5) | 0 (0.0) | 0 (0.0) |
| Exact Age at Five-Year Visit (years) | 5.0 [5.0, 5.1] | 5.0 [5.0, 5.1] | 8.4 [8.1, 8.8] |
| Missing | 658 (19.1) | 0 (0.0) | 0 (0.0) |
| Physical Activity at Five-Year Visit (hours/week) | 2.0 [1.0, 3.0] | 2.0 [1.0, 3.0] | 7.0 [5.0, 12.0] |
| Missing | 1218 [35.3] | 121 (5.9) | 208 (13.2) |

Values are means ± SD for continuous variables with a normal distribution, numbers (%) for categorical variables, or medians [IQR] for continuous variables with a skewed distribution based on non-imputed data. BMI = body mass index.

## **Supplementary Table 3. Descriptive characteristics of participants enrolled in the study stratified across milk fat content categories at age five (N= 2043)**

|  | **No Fat (Skim) (N= 88)** | **1% Fat**  **(N= 466)** | **2% Fat**  **(N= 1000)** | **3.25% Fat**  **(N= 489)** |
| --- | --- | --- | --- | --- |
| **Family Characteristics** |  |  |  |  |
| Maternal Post-Secondary Education (Yes vs. No) | 75 (85.4) | 368 (79.0) | 786 (78.6) | 381 (77.9) |
| Maternal BMI (kg/m²) | 25.3 (4.3)† | 25.4 (5.82)† | 24.9 (5.6)† | 23.7 (4.6)† |
| Other Siblings (Yes vs. No) | 59 (67.0)† | 247 (53.0)† | 475 (47.5)† | 188 (38.4)† |
| Study Site |  |  |  |  |
| Edmonton | 27 (30.7)† | 129 (27.7)† | 226 (22.6)† | 96 (19.6)† |
| Manitoba | 28 (31.8)† | 187 (40.1)† | 308 (30.8)† | 120 (24.5)† |
| Toronto | 21 (23.9)† | 78 (16.7)† | 251 (25.1)† | 97 (19.8)† |
| Vancouver | 12 (13.6)† | 72 (15.5)† | 215 (21.5)† | 176 (36.0)† |
| **Birth Characteristics** |  |  |  |  |
| Child Sex (Males vs. Females) | 45 (51.1) | 246 (52.8) | 508 (50.8) | 277 (56.6) |
| Birth Weight (kg) | 3.5 (0.5) | 3.5 (0.5) | 3.5 (0.5) | 3.4 (0.5) |
| Child Ethnicity |  |  |  |  |
| Caucasian White | 67 (76.1)† | 384 (82.4)† | 657 (65.7)† | 252 (51.5)† |
| Multiracial | 19 (21.6)† | 64 (13.7)† | 237 (23.7)† | 142 (29.0)† |
| Other | 2 (2.3)† | 18 (3.9)† | 106 (10.6)† | 95 (19.4)† |
| Exclusive Breastfeeding at 6 Months (Yes vs. No) | 19.2 (21.6)† | 85 (18.2)† | 165 (16.5)† | 111 (22.7)† |
| **Childhood Characteristics** |  |  |  |  |
| Daily Caloric Intake at Dietary Assessment (kcal/day) | 1516.6  [1182.4, 1818.8] | 1494.8  [1204.4, 1793.6] | 1508.6  [1249.1, 1822.3] | 1490.6  [1225.4, 1818.7] |
| Volume of Milk Intake (cup/day) | 0.5 [0.4, 1.3] | 0.5 [0.4, 1.3] | 0.5 [0.4, 1.3] | 0.5 [0.4, 1.3] |
| Volume of Sugar-Sweetened Beverage Intake (cup/day) | 0.1 [0.0, 0.4] | 0.1 [0.0, 0.3] | 0.1 [0.0, 0.4] | 0.1 [0.0, 0.4] |
| Exact Age at Outcome Assessment (years) | 5.0 [5.0, 5.1] | 5.0 [5.0, 5.1] | 5.0 [5.0, 5.1] | 5.0 [5.0, 5.1] |
| Physical Activity (hours/week) at Age Five | 2.0 [1.1, 4.0] | 2.0 [1.0, 3.0] | 2.0 [1.0, 3.0] | 2.0 [1.0, 3.0] |
| Body Mass Index Z-score at Age Five | 0.6 (1.1)† | 0.5 (0.9)† | 0.4 (1.0)† | 0.2 (0.9)† |
| Waist to Height Z-score at Age Five | -0.2 (1.0)† | -0.2 (0.9)† | -0.3 (1.0)† | -0.5 (1.0)† |
| Obesity Status (Yes vs. No) at Age Five | 6 (6.8)† | 22 (4.7)† | 51 (5.1)† | 9 (1.8)† |

Values are means ± SD for continuous variables with a normal distribution, numbers (%) for categorical variables, or medians [IQR] for continuous variables with skewed distribution based on non-imputed data. Univariate tests were done to compare characteristics among participants across milk fat content levels, using a one-way ANOVA for normally distributed variables, a Kruskal-Wallis test for non-normally distributed variables, and chi-square test for categorical variables. † p-value < 0.05. BMI = body mass index.

## **Supplementary Figure 2: Associations of milk fat content at age five, adiposity indicators and obesity status at age five from basic adjusted analyses (N= 2043)**


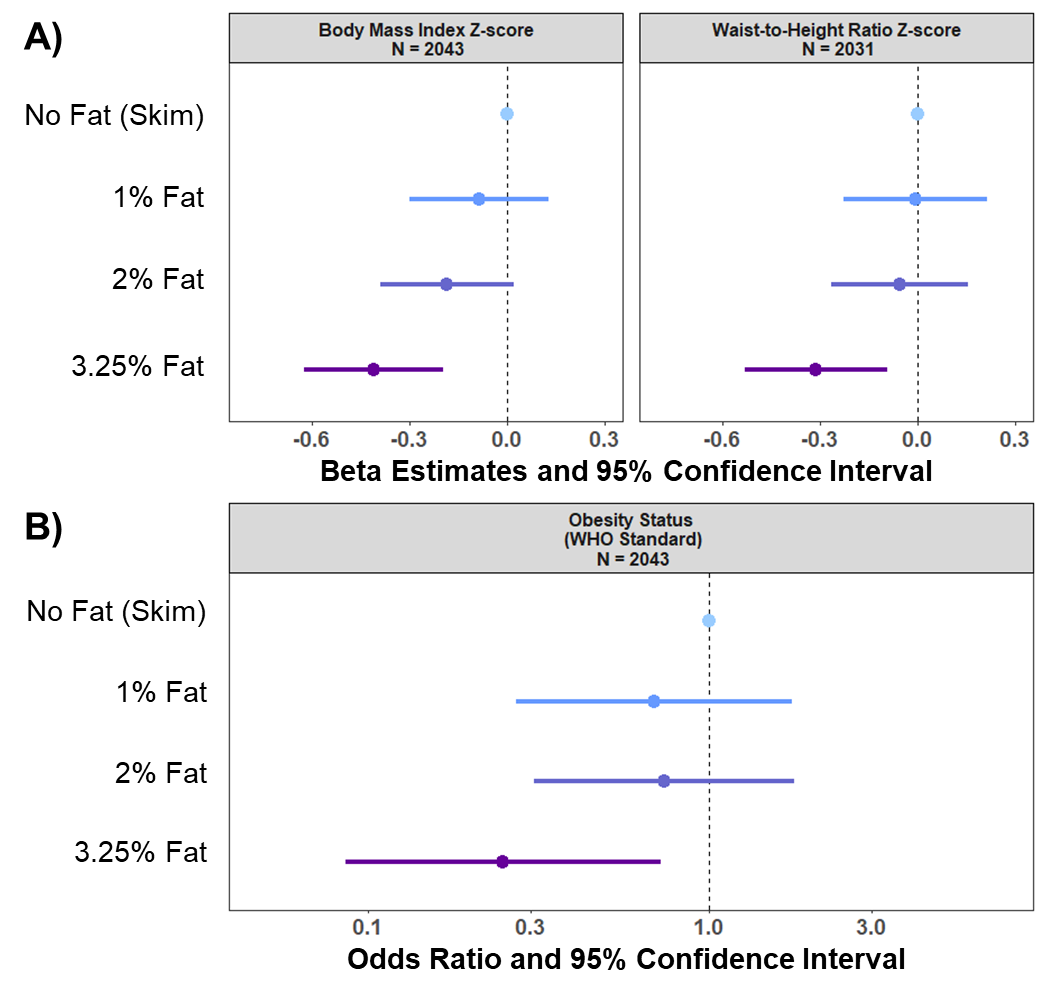


Values are: (A) β estimates representing standardized mean differences with 95% confidence intervals from linear regression analyses for body mass index (BMI) z-score and waist-to-height z-score, for children in a given milk-fat category compared with the reference group (skim milk) at age five; (B) odds ratios and 95% confidence intervals from logistic regression analysis of milk fat content at age five and obesity status at age five. These models account for energy intake at five years of age.

## **Supplementary Figure 3: Associations of milk fat content at age five, adiposity indicators and obesity status at age five, additionally accounting for change in BMI from ages three to five (N= 1960)**

**
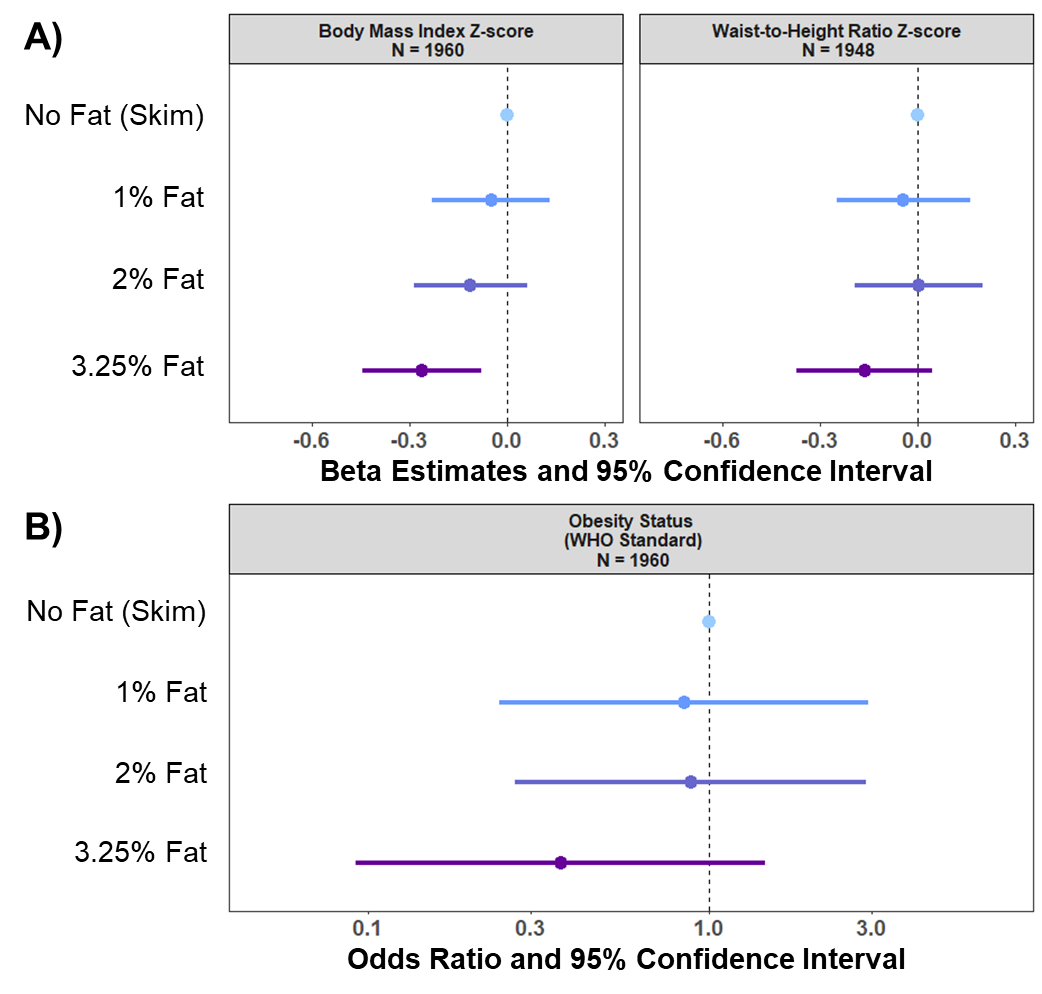
**

Values are: (A) β estimates representing standardized mean differences with 95% confidence intervals from linear regression analyses for body mass index (BMI) z-score and waist-to-height z-score, for children in a given milk-fat category compared with the reference group (skim milk) at age five; (B) odds ratios and 95% confidence intervals from logistic regression analysis of milk fat content at age five and obesity status at age five. The multivariable-adjusted analyses account for maternal BMI, maternal post-secondary education level, child’s race, child’s birthweight, breastfeeding exclusivity, having older siblings, energy intake at five years of age, sugar-sweetened beverage consumption at five years of age, milk consumption at five years of age, organized physical activity at age five, change in BMI from ages three to five and study center site. WHO = World Health Organization.

## **Supplementary Figure 4: Associations of milk fat content at age five, adiposity indicators and obesity status at age five, additionally accounting for total saturated fat intake at age three (N= 2043)**


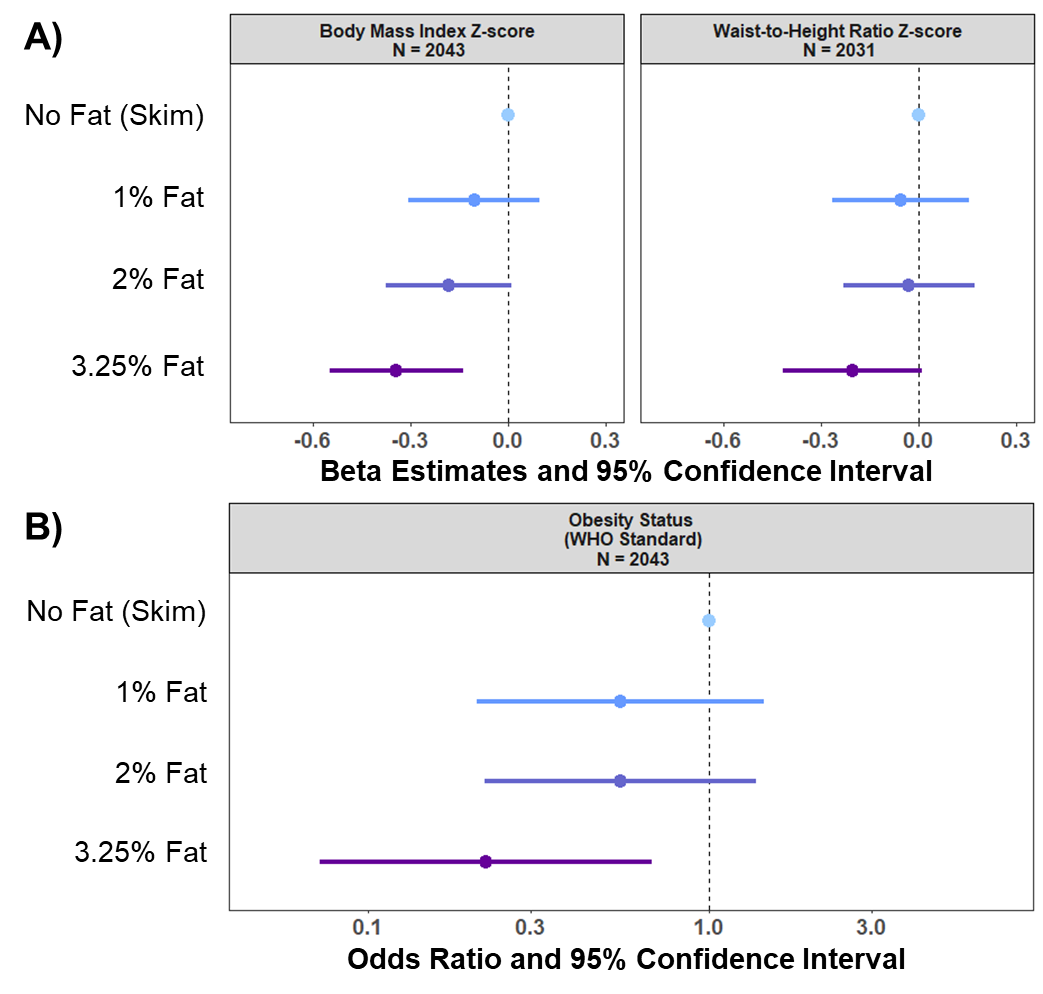


Values are: (A) β estimates representing standardized mean differences with 95% confidence intervals from linear regression analyses for body mass index (BMI) z-score and waist-to-height z-score, for children in a given milk-fat category compared with the reference group (skim milk) at age five; (B) odds ratios and 95% confidence intervals from logistic regression analysis of milk fat content at age five and obesity status at age five. The multivariable-adjusted analyses account for maternal BMI, maternal post-secondary education level, child’s race, child’s birthweight, breastfeeding exclusivity, having older siblings, energy intake at five years of age, sugar-sweetened beverage consumption at five years of age, milk consumption at five years of age, organized physical activity at age five, total saturated fat intake at five years, and study center site. WHO = World Health Organization.

## **Supplementary Figure 5: Associations of milk fat content at age five, adiposity indicators and obesity status at age five, with 1% milk fat as the reference (N= 2043)**

**
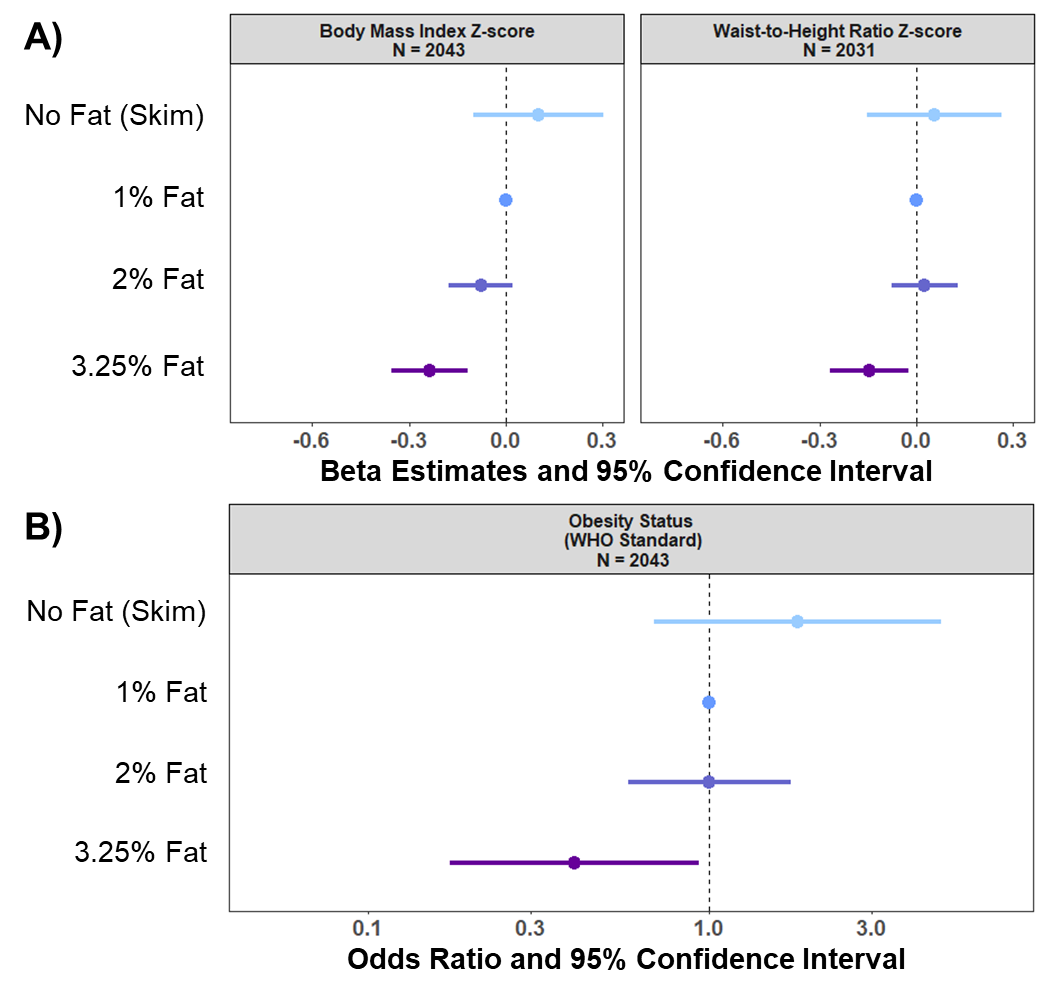
**

Values are: (A) β estimates representing standardized mean differences with 95% confidence intervals from linear regression analyses for body mass index (BMI) z-score and waist-to-height z-score, for children in a given milk-fat category compared with the reference group (1% milk) at age five; (B) odds ratios and 95% confidence intervals from logistic regression analysis of milk fat content at age five and obesity status at age five. The multivariable-adjusted analyses account for maternal BMI, maternal post-secondary education level, child’s race, child’s birthweight, breastfeeding exclusivity, having older siblings, energy intake at five years of age, sugar-sweetened beverage consumption at five years of age, milk consumption at five years of age, organized physical activity at age five, and study center site. WHO = World Health Organization.

## **Supplementary Figure 6: Associations of milk fat content at age five and adiposity indicators at age five, restricted among participants with obesity data at both time points (N= 1545)**

**
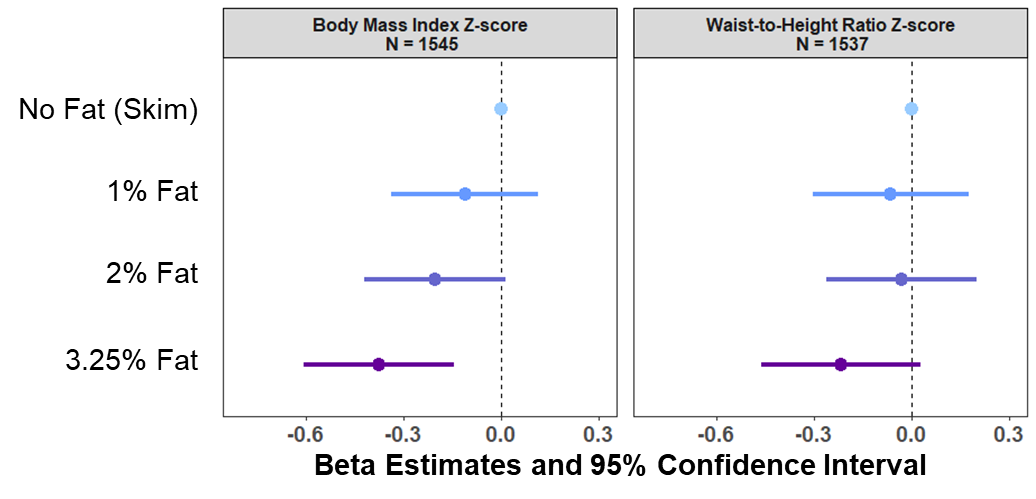
**

Values are β estimates representing standardized mean differences with 95% confidence intervals from linear regression analyses for body mass index (BMI) z-score and waist-to-height z-score, for children in a given milk-fat category compared with the reference group (skim milk) at age five. The multivariable-adjusted analyses account for maternal BMI, maternal post-secondary education level, child’s race, child’s birthweight, breastfeeding exclusivity, having older siblings, energy intake at five years of age, sugar-sweetened beverage consumption at five years of age, milk consumption at five years of age, organized physical activity at age five and study center site.

## **Supplementary Figure 7: Associations of milk fat content at age five, adiposity indicators and obesity status at age eight from basic adjusted analyses (N= 1574)**


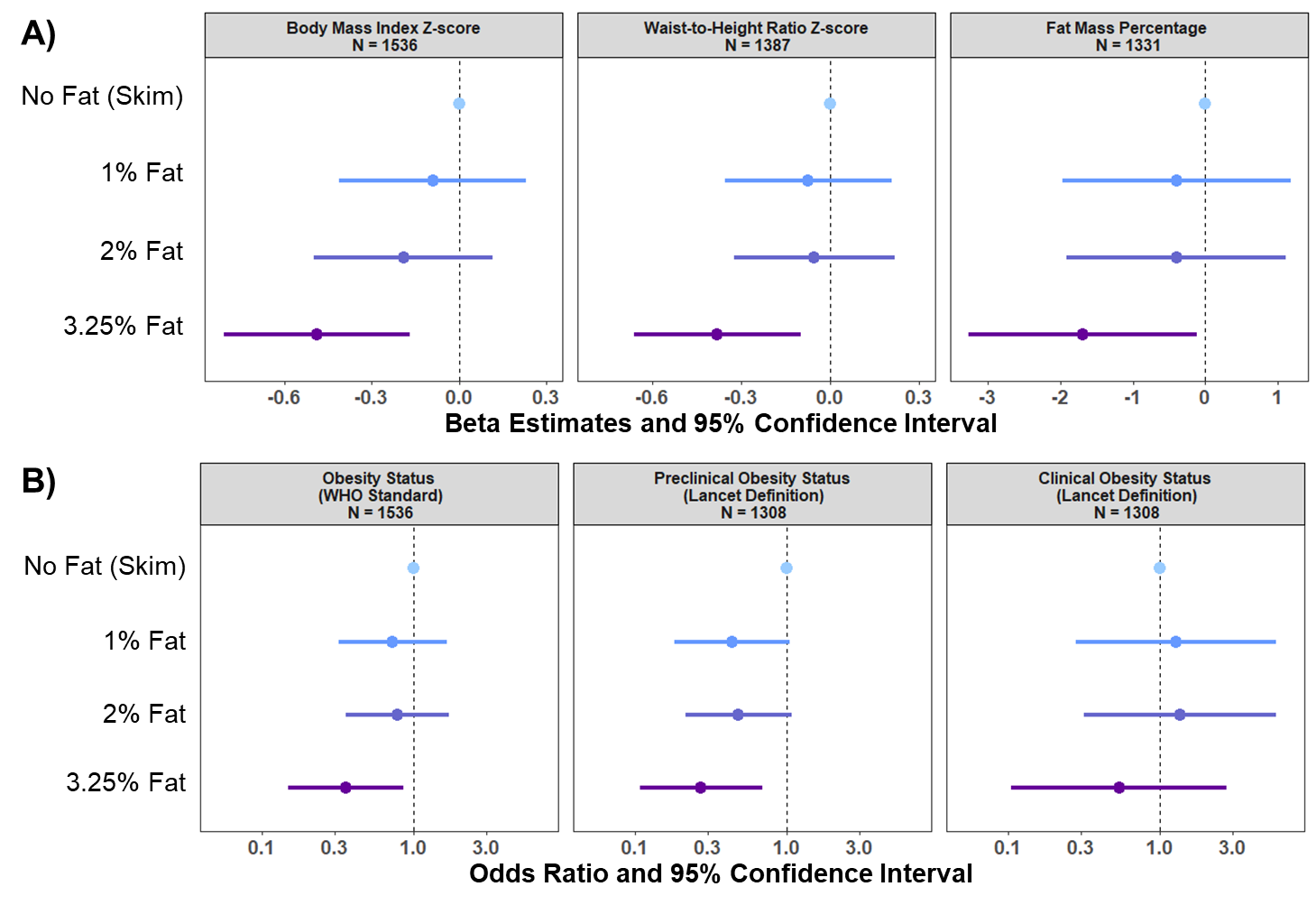


Values are: (A) β estimates representing standardized mean differences with 95% confidence intervals from linear regression analyses for children in a given milk-fat category compared with the reference group (skim milk) at age five for body mass index (BMI) z-score and waist-to-height z-score, and the mean difference in fat mass percentage at age eight; (B) odds ratios and 95% confidence intervals from logistic regression analysis of milk fat content at age five and obesity status at age eight. These models account for energy intake at five years of age for all outcomes, and age and sex were accounted for in the fat mass percentage model. WHO = World Health Organization.

## **Supplementary Figure 8: Associations of milk fat content at age five, adiposity indicators and obesity status at age eight, additionally accounting for change in BMI from ages three to five (N= 1486)**


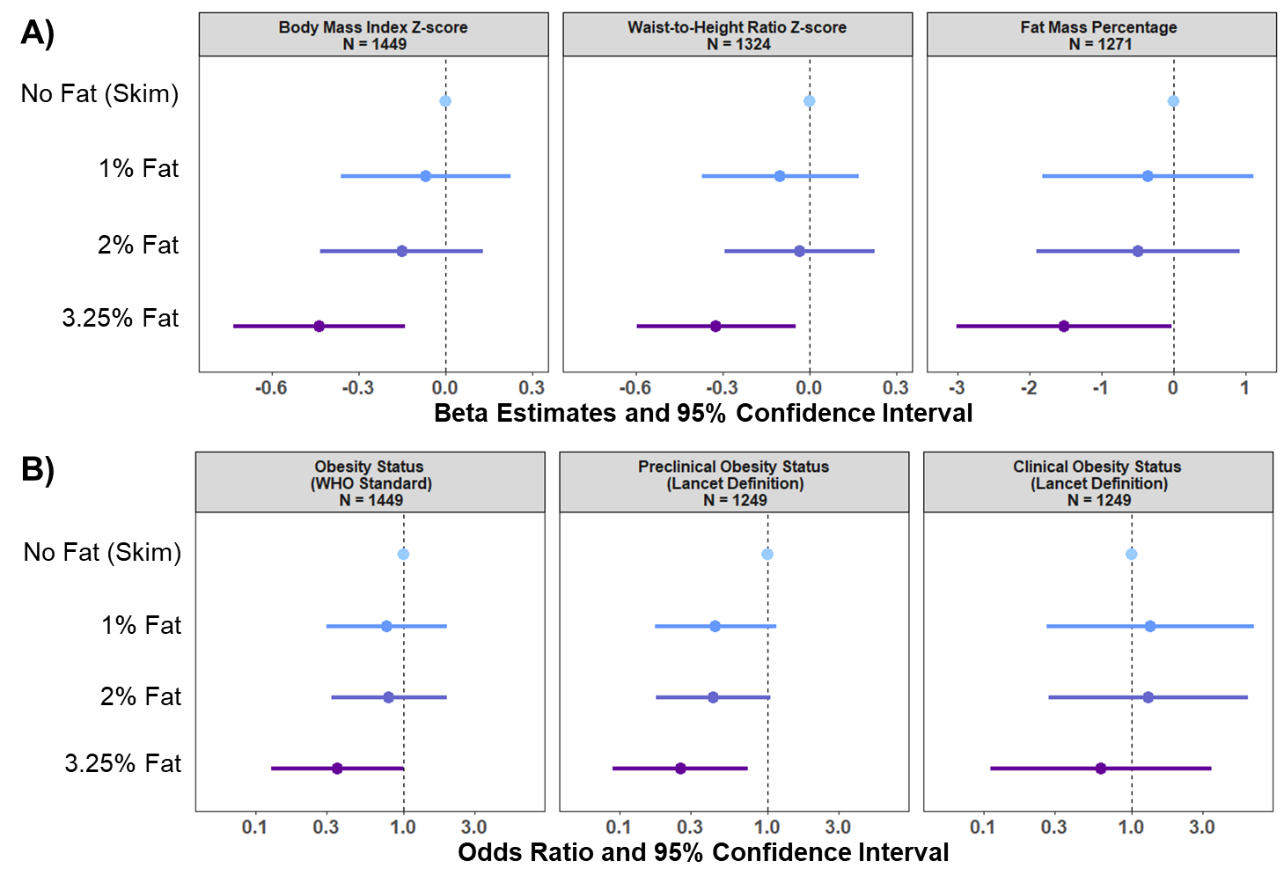


Values are: (A) β estimates representing standardized mean differences with 95% confidence intervals from linear regression analyses for children in a given milk-fat category compared with the reference group (skim milk) at age five for body mass index (BMI) z-score and waist-to-height z-score, and the mean difference in fat mass percentage at age eight; (B) odds ratios and 95% confidence intervals from logistic regression analysis of milk fat content at age five and obesity status at age eight. The multivariable-adjusted analyses account for maternal BMI, maternal post-secondary education level, child’s race, child’s birthweight, breastfeeding exclusivity, having older siblings, energy intake at five years of age, sugar-sweetened beverage consumption at five years of age, milk consumption at five years of age, organized physical activity at age eight, modified alternative healthy eating index (mAHEI) score at age eight, change in BMI from ages three to five and study center site. WHO = World Health Organization.

## **Supplementary Figure 9: Associations of milk fat content at age five, adiposity indicators and obesity status at age five, additionally accounting for total saturated fat intake at age three (N= 1574)**


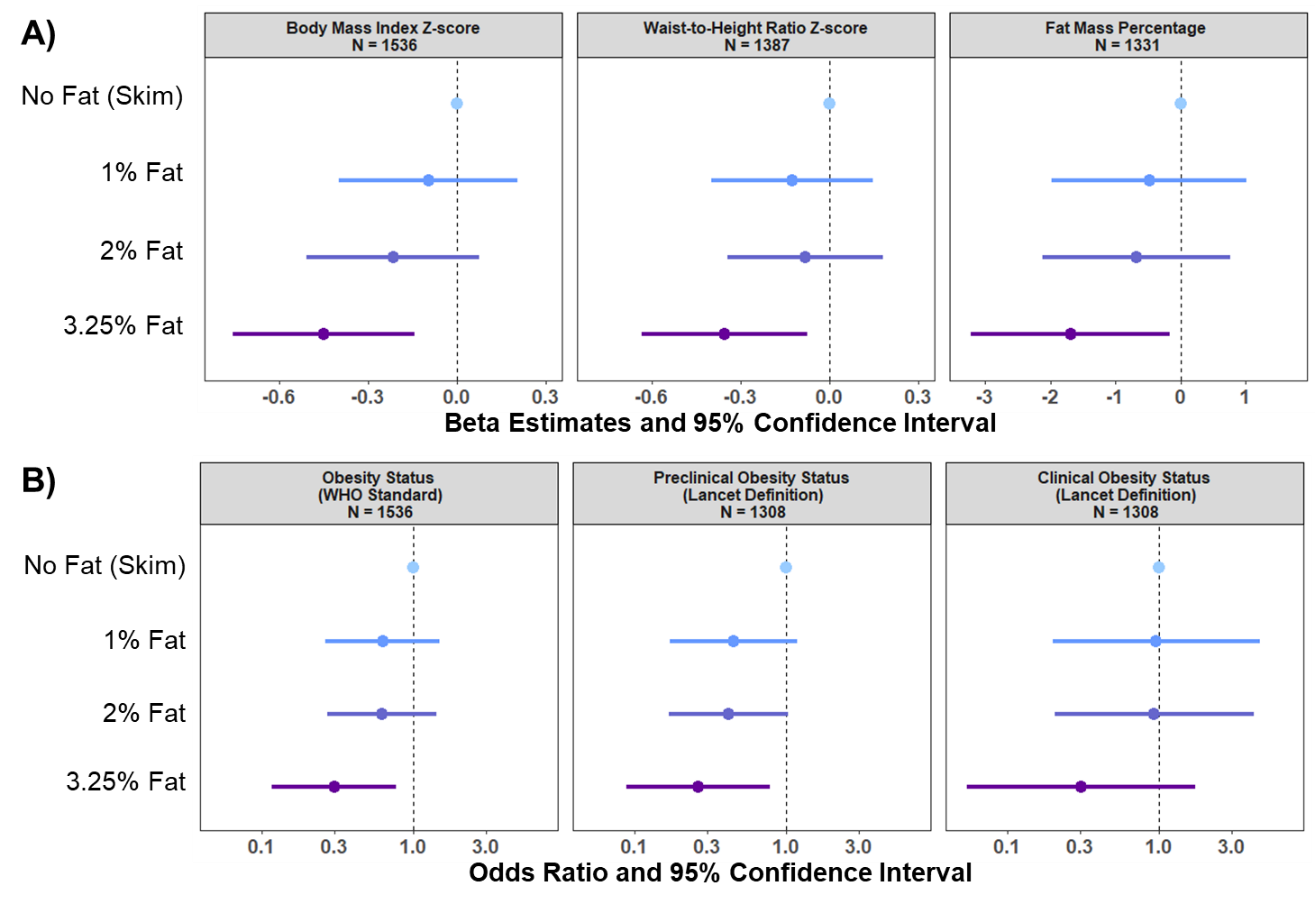


Values are: (A) β estimates representing standardized mean differences with 95% confidence intervals from linear regression analyses for children in a given milk-fat category compared with the reference group (skim milk) at age five for body mass index (BMI) z-score and waist-to-height z-score, and the mean difference in fat mass percentage at age eight; (B) odds ratios and 95% confidence intervals from logistic regression analysis of milk fat content at age five and obesity status at age eight. The multivariable-adjusted analyses account for maternal BMI, maternal post-secondary education level, child’s race, child’s birthweight, breastfeeding exclusivity, having older siblings, energy intake at five years of age, sugar-sweetened beverage consumption at five years of age, milk consumption at five years of age, modified alternative healthy eating index (mAHEI) score, organized physical activity at age eight, total saturated fat intake at five years, and study center site. WHO = World Health Organization.

## **Supplementary Figure 10: Associations of milk fat content at age five, adiposity indicators and obesity status at age eight, accounting for dairy intake at age eight (N= 1574)**


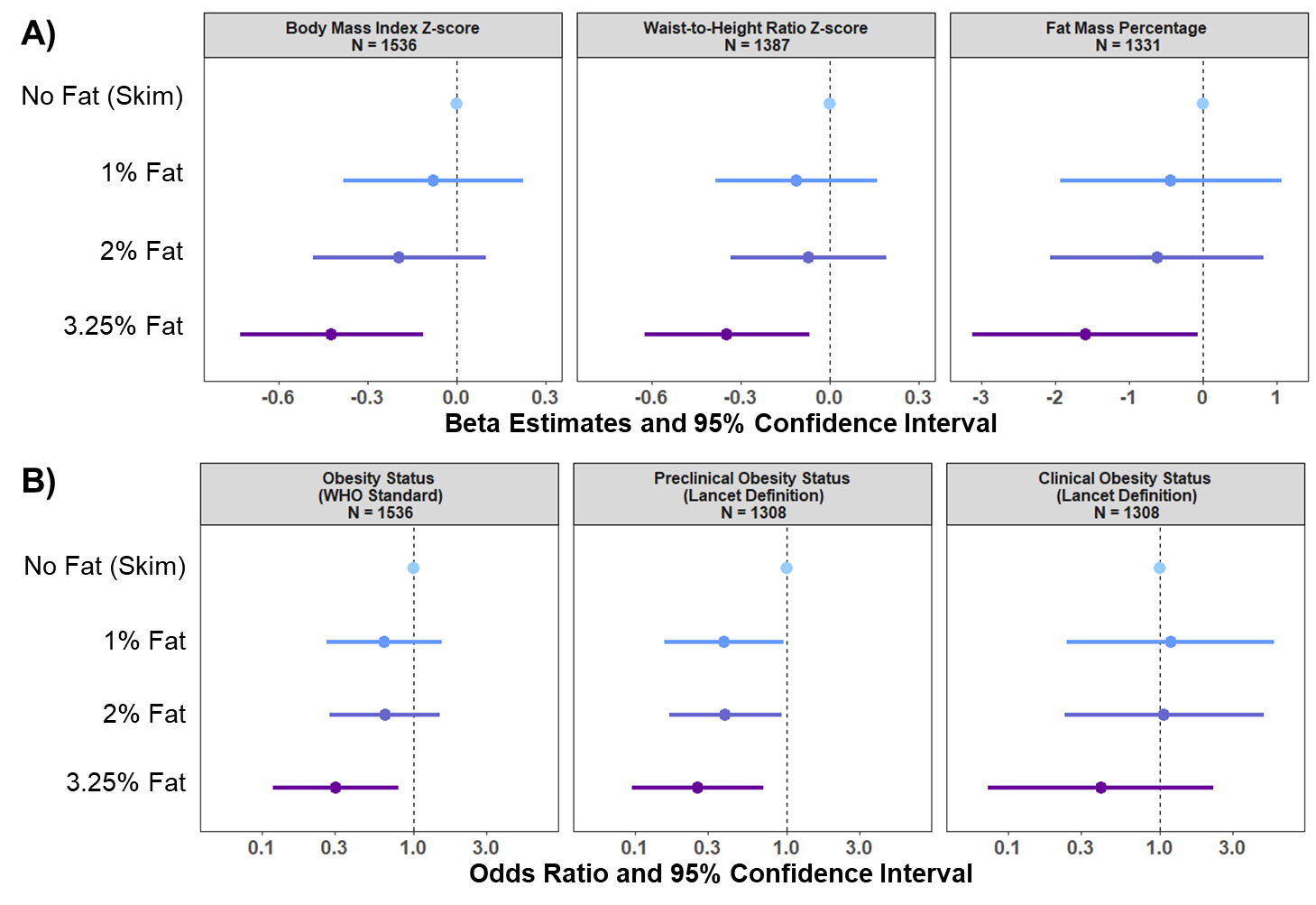


Values are: (A) β estimates representing standardized mean differences with 95% confidence intervals from linear regression analyses for children in a given milk-fat category compared with the reference group (skim milk) at age five for body mass index (BMI) z-score and waist-to-height z-score, and the mean difference in fat mass percentage at age eight; (B) odds ratios and 95% confidence intervals from logistic regression analysis of milk fat content at age five and obesity status at age eight. The multivariable-adjusted analyses account for maternal BMI, maternal post-secondary education level, child’s race, child’s birthweight, breastfeeding exclusivity, having older siblings, energy intake at five years of age, sugar-sweetened beverage consumption at five years of age, milk consumption at five years of age, organized physical activity at age eight, modified alternative healthy eating index (mAHEI) score at age eight, dairy intake at age eight, and study center site. WHO = World Health Organization.

## **Supplementary Figure 11: Associations of milk fat content at age five, adiposity indicators and obesity status at age eight, with 1% milk fat as the reference (N= 1574)**


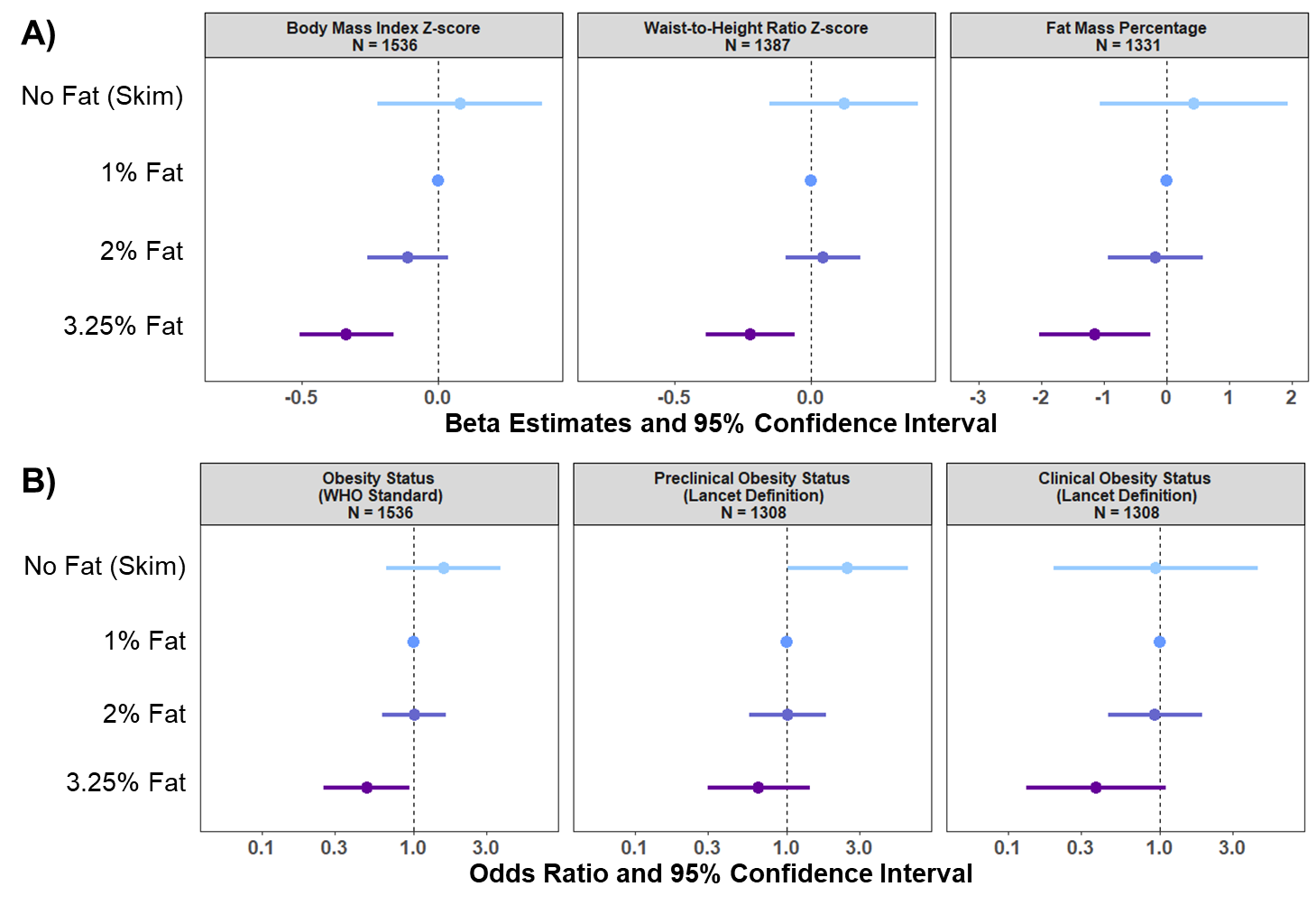


Values are: (A) β estimates representing standardized mean differences with 95% confidence intervals from linear regression analyses for children in a given milk-fat category compared with the reference group (1% milk) at age five for body mass index (BMI) z-score and waist-to-height z-score, and the mean difference in fat mass percentage at age eight; (B) odds ratios and 95% confidence intervals from logistic regression analysis of milk fat content at age five and obesity status at age eight. The multivariable-adjusted analyses account for maternal BMI, maternal post-secondary education level, child’s race, child’s birthweight, breastfeeding exclusivity, having older siblings, energy intake at five years of age, sugar-sweetened beverage consumption at five years of age, milk consumption at five years of age, organized physical activity at age eight, modified alternative healthy eating index (mAHEI) score at age eight and study center site. WHO = World Health Organization.

## **Supplementary Figure 12: Associations of milk fat content age five and adiposity indicators at age eight, restricted among participants with obesity data at both time points (N= 1545)**


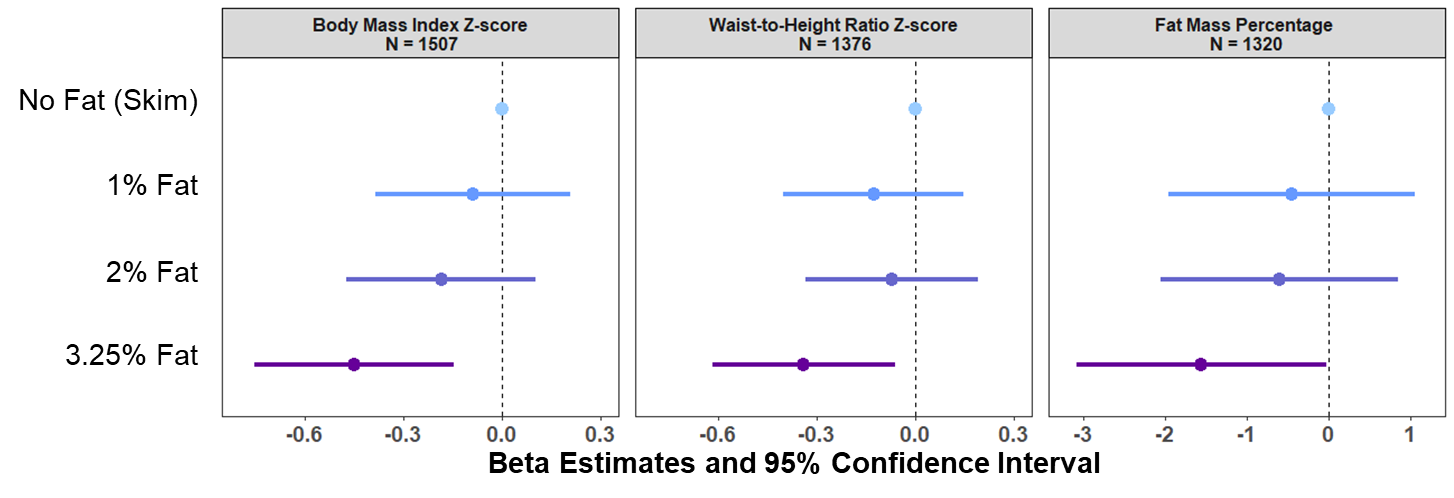


Values are β estimates representing standardized mean differences with 95% confidence intervals from linear regression analyses for children in a given milk-fat category compared with the reference group (skim milk) at age five for body mass index (BMI) z-score and waist-to-height z-score, and the mean difference in fat mass percentage at age eight. The multivariable-adjusted analyses account for maternal BMI, maternal post-secondary education level, child’s race, child’s birthweight, breastfeeding exclusivity, having older siblings, energy intake at five years of age, sugar-sweetened beverage consumption at five years of age, milk consumption at five years of age, organized physical activity at age eight, modified alternative healthy eating index (mAHEI) score at age eight and study center site.
